# Factors That Influence Career Choice among Different Populations of Neuroscience Trainees

**DOI:** 10.1523/ENEURO.0163-21.2021

**Published:** 2021-06-18

**Authors:** Lauren E. Ullrich, John R. Ogawa, Michelle D. Jones-London

**Affiliations:** Office of Programs to Enhance Neuroscience Workforce Diversity, National Institute of Neurological Disorders and Stroke, National Institutes of Health, Bethesda, Maryland 20892

**Keywords:** career choice, diversity, workforce

## Abstract

Specific groups have historically been, and continue to be, underrepresented in the biomedical research workforce, especially academia. Career choice is a multifactorial process that evolves over time; among all trainees, expressed interest in faculty research careers decreases over time in graduate school, but that trend is amplified in women and members of historically underrepresented racial and ethnic groups (Golde and Dore, 2004; Fuhrmann et al., 2011; Sauermann and Roach, 2012; Gibbs et al., 2014; Roach and Sauermann, 2017). This work was designed to investigate how career interest changes over time among recent neuroscience PhD graduates, and whether differences in career interests are associated with social identity, experiences in graduate school and postdoctoral training, and personal characteristics. We report results from a survey of 1479 PhD neuroscientists (including 16% underrepresented scientists and 54% women scientists). We saw repeated evidence that individual preferences about careers in general, and academic careers specifically, predict current career interest. These findings were moderated by social identity and experiences in graduate school and postdoctoral training. Our findings highlight the important influence of the advisor in shaping a trainee’s career path, and the ways in which academic culture is perceived as unwelcoming or incongruent with the values or priorities of certain groups. They suggest several areas for positive growth, ways to change how we think about the impact of mentorship, and policy and programmatic interventions that extend beyond trying to change or “fix” the individual and instead recognize the systemic structures that influence career choices.

## Significance Statement

Specific groups have historically been, and continue to be, underrepresented in the biomedical research workforce. Career choice is a multifactorial process that evolves over time; among all trainees, expressed interest in faculty research careers decreases over time, but that trend is amplified in women and members of historically underrepresented racial and ethnic groups. A survey of 1479 PhD neuroscientists revealed evidence that preferences about careers in general, and academic careers specifically, affect career interest and are moderated by social identity and experiences in graduate and postdoctoral training. Our findings suggest areas for positive growth, ways to rethink the impact of mentorship, and interventions that extend beyond “fixing” the individual and instead recognize the systemic structures that influence career choices.

## Introduction

During the past few decades, the biomedical sciences career track has undergone significant changes. The number of doctorate recipients in the life sciences has nearly doubled in the past 30 years, while the number of tenure-track faculty appointments 3–5 years after graduation has remained flat (Larson et al., 2014; Roach and Sauermann, 2017). Consistent with this trend, the share of life science PhDs holding faculty positions has declined: in 1993, 17.3% of life science PhD graduates held a tenure-track position 3–5 years after graduation; by 2013, that percentage was 10.6% (National Science Board, 2016). This sea change in career prospects and outcomes has sparked a national conversation within the scientific community about how trainees make career choices and how to best prepare them for their future careers (National Institutes of Health, 2012; Gibbs and Griffin, 2013; Gibbs et al., 2014; Sinche, 2016).

As a discipline, neuroscience has characteristics that may exacerbate the overall trends seen in the life sciences. For example, the number of trainees in neuroscience has been expanding much more rapidly than in other fields. Between 1990 and 2013, the number of neuroscience PhDs awarded increased more than fivefold—in comparison, the number of biology PhDs awarded increased twofold [see 2016 Survey of Earned Doctorates (https://www.nsf.gov/statistics/2018/nsf18304/)]. Consequently, the magnitude of the difference between the number of trainees and faculty positions in neuroscience is likely greater than in biology and biomedicine overall. This is supported by the fact that in 2017, 5% of neurobiology and neuroscience PhD holders who were 3–7 years after graduation held a tenured or tenure-track position, compared with 10% of comparable biology, agriculture, and environmental life science PhDs as a group [calculated from data in the National Science Foundation (NSF) National Survey of College Graduates, PUBLIC 2019 (https://ncsesdata.nsf.gov/sestat/)]. This may lead students and/or faculty to place more emphasis on preparation for careers other than research faculty positions. An example of a program created to respond to this trend is the National Institutes of Health (NIH) Broadening Experiences in Scientific Training initiative, which prepares individuals for a broader range of careers in the biomedical research enterprise (Lenzi et al., 2020). This kind of program presents alternatives to trainees who are currently spending longer time periods as postdoctoral fellows competing for limited faculty positions.

Historically, specific groups have been, and continue to be, underrepresented (UR) in science and technology. These underrepresented groups include American Indians/Alaska Natives; Blacks/African Americans; Hispanics/Latinos; Native Hawaiians/Other Pacific Islanders; and persons with disabilities [see 2016 Survey of Earned Doctorates (https://www.nsf.gov/statistics/2018/nsf18304/)]. These groups are underrepresented at every level of postsecondary education, and underrepresentation is progressively greater at every rung on the academic ladder (National Academies of Sciences, Engineering, and Medicine, 2011). For example, in 2016, scientists that belong to underrepresented racial and ethnic groups received 14% of life science doctoral degrees, but made up only 10% of tenured and tenure-track life science faculty in the United States [see 2015 Survey of Doctorate Recipients (https://ncsesdata.nsf.gov/doctoratework/2015/) and 2016 Survey of Earned Doctorates (https://www.nsf.gov/statistics/2018/nsf18304/)].

Enhancing the diversity of the scientific workforce is critical to “fostering scientific innovation, enhancing global competitiveness, contributing to robust learning environments, improving the quality of the research, advancing the likelihood that underserved or health disparity populations participate in, and benefit from health research, and enhancing public trust” [see Notice of NIH's Interest in Diversity, Notice Number, NOT-OD-20-031 (https://grants.nih.gov/grants/guide/notice-files/NOT-OD-20-031.html)]. However, decades of efforts by the NIH and others to “enhance the pipeline” by increasing the entry of women and underrepresented scientists into undergraduate and PhD science programs has not had an appreciable effect on the relative proportion of underrepresented tenure-track faculty (Gibbs et al., 2016). This has commonly been described as “the leaky pipeline” (Miller and Wai, 2015), a metaphor that assumes that at PhD entry all trainees aspire to a faculty research position, but some “leak” from the faculty pipeline into a different career. The framing has evolved over time from a discussion about the “pipeline” to a recognition of different “pathways” to science (see https://www.higheredtoday.org/2016/02/10/reconsidering-the-pipeline-problem-increasing-faculty-diversity/). It is possible that some trainees either never wanted a faculty research position or were interested in a variety of professions. Alternatively, trainees may be or feel forced out of the faculty track by a lack of opportunities, an unwelcoming academic culture, or other circumstances beyond their control.

Moreover, compared with well represented students, women and people from underrepresented groups (including and perhaps especially those with multiple underrepresented or marginalized identities) face additional or unique challenges in training, and may make different career choices based on their experiences and values (Sauermann and Roach, 2012; Gibbs and Griffin, 2013; Fisher et al., 2019). Career choice is a multifactorial process that evolves over time; among all trainees, expressed interest in faculty research careers decreases over time in graduate school, but that trend is amplified in women and members of US-based historically underrepresented racial and ethnic groups (Golde and Dore, 2004; Fuhrmann et al., 2011; Sauermann and Roach, 2012; Gibbs et al., 2014; Roach and Sauermann, 2017). Previous research has shown that factors such as research self-efficacy (confidence in one's ability as a researcher); social and intellectual feeling of belonging; and interactions with one’s advisor can all affect career interests, particularly among underrepresented scientists (Estrada et al., 2011, 2019; Gazley et al., 2014; Gibbs et al., 2014; Hayter and Parker, 2019).

To create and administer effective training programs for a diverse research workforce, in 2017, the National Institute of Neurologic Disorders and Stroke (NINDS) sought information about the factors influencing career choice among different populations, particularly those underrepresented in the neuroscience workforce. This work was designed to investigate how career interest changes over time among recent neuroscience PhD graduates, and whether differences in career interests are associated with social identity (i.e., gender and race/ethnicity), experiences in graduate school and postdoctoral training (e.g., relationship with advisor; feelings of belonging), and personal characteristics (e.g., confidence in one’s potential to be an independent researcher). NINDS sought input from current or recent trainees in the neuroscience field to help inform future training programs and initiatives to better serve the neuroscience community. While the COVID-19 pandemic has created unprecedented challenges worldwide and provided opportunities for long overdue public conversations about structural racism that have affected neuroscientists at all career stages, this survey is a snapshot in time and does not capture the current conditions. However, the academic culture and systemic training environments highlighted in the survey remain relevant. NINDS is committed to the development of a biomedical research workforce that is representative of the diversity in American society, and the information collected from this study was aimed to help give NINDS and the entire neuroscience community a clearer picture of the environment and experiences of our trainee and potential trainee community.

## Materials and Methods

### Sample

The study population was composed of (1) recent doctoral recipients [calendar year 2008 (CY2008) or later] who were (2) US citizens or permanent residents and (3) had applied for NINDS funding or had been appointed to NINDS training (T32) or applied for research education grants (R25). In addition to capturing post-trainees across a decade, the year 2008 was chosen as a cutoff because 2003 marked a clear turning point in NIH funding: between 1998 and 2003, the NIH budget almost doubled, whereas from 2003 to 2017, when this survey was conducted, NIH funding plateaued in real dollars and decreased in relative purchasing power [FASEB Office of Public Affairs, 2020; see also National Institutes of Health Funding: FY1995-FY2021 (https://fas.org/sgp/crs/misc/R43341.pdf)]. Those who were in graduate school between 2003 and the present likely had very different experiences than those who entered graduate school earlier than 2003. Since the average time to a neuroscience PhD is 5–6 years (Lorden et al., 2011), those graduating in 2008 entered around 2003—hence, the choice of 2008 as the cutoff point.

Potential participants for this study were identified within the NIH Information for Management Planning Analysis and Coordination II (IMPACII) database, a database containing administrative data from all extramural grant applications (Institute of Medicine Council on Health Care Technology, 1988). A total of 7405 eligible or likely eligible individuals were identified in IMPACII through these searches. Citizenship and year of PhD conferral information were available for some, but not all, individuals, so not all identified individuals were eligible; respondents were screened for eligibility according to the three criteria above at the beginning of the survey. An e-mail list containing every e-mail address available in the IMPACII system for the 7405 identified individuals was created to allow e-mail outreach for the survey. Approximately half of US citizen or permanent resident neuroscience PhD recipients are supported by NIH during their PhD [see NIH Data Book Report 268 (https://report.nih.gov/nihdatabook/report/268)]. We do not have exact data on the proportion supported by NINDS or the proportion that apply for NINDS funding but do not receive it; both types of individuals were eligible for this survey. As determined by the NIH Office of Human Subjects Research, federal regulations for the protection of human subjects do not apply to this activity.

### Dissemination and data collection

Unique survey invitations were sent on May 10, 2017, through SurveyMonkey (http://www.surveymonkey.com) to all identified e-mail addresses (9758 addresses; an average of 1.3 e-mail addresses per person). Follow-up invitations for those who had not responded were sent through SurveyMonkey every 2 weeks. For emails that were undeliverable or “bounced,” an attempt was made to find a current e-mail address through online searches. Additionally, mentors of eligible F31 and F32 applicants were asked to forward information about the survey to their trainees’ current e-mail addresses. If individuals independently inquired about the survey, eligibility was confirmed before sending a survey invitation.

All participants consented to participation in the study. All survey responses were anonymous. At survey close, on July 1, 2017, a total of 5935 e-mails (61%) had been opened, and 3823 e-mails (39%) were undeliverable or unopened. The survey received 2675 responses (∼36% of identified individuals). Of these responses, 2310 were complete, 250 were ineligible, and 115 gave a partial response. Of the 2310 complete and eligible responses, 65 were from participants who filled out the survey more than once. For multiple responses from the same participant, only the first response was kept, for a final total of 2242 complete, eligible, and unique responses.

### Definitions and sample refinement

Several other criteria were applied to further refine the sample for analysis. First, since gender and race/ethnicity were of primary interest for this article, the responses that did not include that information were excluded, leaving 2065 responses. These responses included all who answered either “male” or “female” and may include transgender respondents who identify as either a man or a woman. Transgender and nonbinary people are estimated to make up ∼0.4% of the population of the United States (Meerwijk and Sevelius, 2017). Accordingly, only two participants indicated “other” and wrote in a response for gender; they were not included in the analysis, because of small numbers. Respondents from white and/or Asian backgrounds are referred to as well represented (WR), while respondents from American Indian/Alaska Native, Black/African American, Hispanic/Latino, and/or Native Hawaiian/Pacific Islander backgrounds are referred to as UR, according to the NSF definition (National Science Foundation, 2015). Second, since the original survey was also aimed at current PhD students, who would not have “end of graduate school” ratings of interest, all current students were dropped, leaving a final sample size of 1479. Disability status was collected, but persons with a disability made up <3% of the sample, so were not included as a separate analysis group because of the small sample size.

### Survey

The survey was a 57-question instrument administered at a single point in time. It asked about respondents’ career interest; experiences in graduate school and postdoctoral training; feelings about careers in general; objective measures of research experience and productivity; and basic demographics. Questions were iteratively developed by synthesizing from several sources, conducting cognitive testing interviews, and refining language where necessary [Gibbs and Griffin, 2013; Griffin et al., 2015; Layton et al., 2016; Malley et al., 2006; Meyers et al., 2016; National Institutes of Health, 2012; Sauermann and Roach, 2012; Sinche, 2016; Sinche et al., 2017; Yoder and Mattheis, 2016; see also Notice of NIH's Interest in Diversity, Notice Number, NOT-OD-20-031 (https://grants.nih.gov/grants/guide/notice-files/NOT-OD-20-031.html); National Postdoctoral Association Core Competencies (https://www.nationalpostdoc.org/page/CoreCompetencies); 2015 Survey of Doctorate Recipients (https://ncsesdata.nsf.gov/doctoratework/2015/); and 2016 Survey of Earned Doctorates (https://www.nsf.gov/statistics/2018/nsf18304/)].

Respondents were asked to rate their interest in pursuing each of the following career pathways at three time points: the start of their PhD program, the end of their PhD program, and currently. These pathways were as follows: academic position, research focus (includes physician-scientist); academic position, teaching focus; nonacademic research (e.g., research in industry, biotech, or government settings); and science-related, nonresearch (e.g., science outreach, communication, policy, advocacy, or administration). Respondents were also asked about their interest in other, non-science-related careers, as this did not measure a specific career path, but a variety of possible careers; and, to reduce the number of variables and analyses, these responses were not analyzed. Interest was measured on a 4-point Likert-type scale where 1 = no interest, 2 = low interest, 3 = moderate interest, and 4 = strong interest.

Respondents were also asked about their social identity (specifically gender and race/ethnicity), experiences in graduate and postdoctoral training, personal characteristics, and objective measures (Extended Data Table 1-1).

Experiences in training included the following: various aspects of their relationship with their primary training advisor during graduate and postdoctoral training (5-point scale from “very negative” to “very positive”); sources and helpfulness of support and career advice during the graduate and postdoctoral training (4-point scale from “no guidance provided” to “very helpful”); feelings of social and intellectual belonging to a laboratory/research group and department/program during graduate and postdoctoral training (5-point scale from “strongly disagree” to “strongly agree”).

Personal characteristics included the following: confidence in one’s potential as an independent researcher (measured on a 5-point agreement scale where 1 was “strongly disagree” and 5 was “strongly agree”); aspects of the career or work environment most important to the respondent (choose up to top 5); and features of academia that increase or decrease desire to become a faculty member (5-point scale from “greatly decrease” to “greatly increase”).

Objective measures included the number of years of research before a PhD program, total number of years of research, number of years to complete a PhD, total time in postdoctoral training, years since PhD completion, support by NIH before the PhD program, first-author publication rate (first-authored publications/total years performing research), time to PhD completion, and undergraduate or doctoral degree from a top 50 research university (as measured by research and development expenditures; National Science Board, 2016). Table 1 presents demographic statistics for the sample.

**Table 1:** Study sample characteristics

Demographic variable		*n* (%)
Gender	Women	793 (54%)
	Men	686 (46%)
UR status	WR	1246 (84%)
	UR	233 (16%)
Social identity	WR women	660 (45%)
	WR men	586 (40%)
	UR women	133 (9%)
	UR men	100 (7%)
Have a disability?	No	1415 (98%)
	Yes	36 (3%)
First person or among the first generation to graduate from a 4 year college?	No	1125 (77%)
	Yes	346 (24%)
Sexual orientation	Straight or heterosexual	1324 (93%)
	Lesbian, gay, homosexual, bisexual, or other sexual minority	87 (7%)
PhD field	Neuroscience	705 (48%)
	Cellular/molecular biology	137 (9%)
	Biological sciences	111 (8%)
	Biochemistry/chemistry	84 (6%)
	Bioengineering	82 (6%)
	Psychology	80 (5%)
	Pharmacology/toxicology	60 (4%)
	Physiology	49 (3%)
	Genetics	34 (2%)
	Biostatistics, epidemiology, public health, clinical sciences	29 (2%)
	Physics	22 (2%)
	Microbiology and immunology	21 (1%)
	Engineering or computer science	20 (1%)
	Pathology	19 (1%)
	Kinesiology	14 (1%)
	Other	12 (1%)
Current position	Postdoc	612 (41%)
	Academic faculty/research	396 (27%)
	Science, nonresearch	150 (10%)
	Research, nonacademic	135 (9%)
	Academic faculty/teaching	101 (7%)
	Nonscience	60 (4%)
	Unemployed	25 (2%)
Carnegie classification of undergraduate institution	Baccalaureate/associate's colleges: mixed baccalaureate/associate's	1 (%)
	Baccalaureate colleges: arts and sciences focus	216 (15%)
	Baccalaureate colleges: diverse fields	28 (2%)
	Special focus 4 year schools	3 (%)
	Master's colleges and universities: small programs	20 (1%)
	Master's colleges and universities: medium programs	34 (2%)
	Master's colleges and universities: larger programs	88 (6%)
	Doctoral/professional universities	13 (1%)
	Doctoral universities: moderate research activity	47 (3%)
	Doctoral universities: high research activity	7 (%)
	Doctoral universities: higher research activity	163 (11%)
	Doctoral universities: highest research activity	703 (48%)
	International/unknown	156 (11%)
Minority serving institution categorization of undergraduate institution	Non-MSI institution	1298 (88%)
	Hispanic serving institution (HSI)	56 (4%)
	Asian American and Pacific Islanders (AAPI) serving institution	48 (3%)
	American Indian and Alaska Native (AIAN) serving institution	37 (3%)
	Historically Black Colleges and Universities (HBCU)	16 (1%)
	HSI and AAPI institution	19 (1%)
	HSI and AIAN institution	5 (%)

Basic demographic information about the sample of 1,479 PhD neuroscientists who responded to the survey. Additional descriptive information for all variables is presented in Extended Data Table 1-1. UR = underrepresented, WR = well represented.

10.1523/ENEURO.0163-21.2021.t1-1 Extended Data Table 1-1 Descriptive information for all variables. Descriptive statistics for all dependent and independent variables in the study, by category of variable. Min = minimum value, Max = maximum value, N = number in group, SD = standard deviation. Download Table 1-1, DOC file.

### Analysis

#### Variable testing and data reduction

This work was designed to investigate how career interest evolves over time, and whether changes and/or differences in career interests are associated with gender and race/ethnicity, experiences in graduate school and postdoctoral training, and personal characteristics. Outcome variables were ratings of interest in the different career types, represented on a 4-point scale, at the following three timepoints: start of PhD program (T1), end of PhD program (T2), and current (T3). All independent (explanatory) variables were split between those used to predict interest at the end of respondents’ PhD programs (e.g., feelings of belonging during PhD) and those used to predict current interest (e.g., feelings of belonging during postdoctoral training). Variables that are the result of factor analysis are indicated in Extended Data Table 1-1.

Dichotomous variables were recoded to 0 and 1. Before any analyses, all continuous variables were visually checked for outliers by plotting and comparing with similar curves, and any outliers were recoded to the largest/smallest value that fit the visual curve (cap method). Using this method, only four observations were capped. Continuous predictor variables were centered when analyzing interactions.

Although the outcome variables are ordinal in nature, it was preferable to treat them as interval in these analyses. Accordingly, we tested their suitability for use as interval variables by using a procedure outlined by Jacoby (1999; see also https://web.archive.org/web/20180827212513/http://polisci.msu.edu/jacoby/software/optiscale/Jacoby,%20opscale%20MS,%203-26-12.pdf). First, the ordinal variables were converted through alternating least-squares optimal scaling optimal scaling to create interval-level representations. Then the interval variables were correlated with the original variables, and we found that a very strong linear relationship existed: correlations ranged from a low of 0.9644 to a high of 0.9997, all significant at the *p* < 0.0001 level. This process was repeated with all other ordinal variables in the study, and correlations ranged from 0.9377 to 0.9991, all significant at the *p* < 0.0001 level. Therefore, we felt comfortable treating all ordinal-level variables as interval-level variables in the analyses.

Finally, data reduction was performed for several constructs to reduce multicollinearity and the problem of multiple comparisons and type I error. We used factor analysis to reduce these constructs (e.g., relationship with advisor) into latent factors.

For each analysis the number of factors to extract was ascertained using Bayesian information criterion scores computed through the VSS function from the psych package (Revelle, 2019) in R. Then, the fa function (also from the psych package) was used to compute maximum-likelihood solutions, with oblique rotation performed using the “promax” option. Thirty-two questions were reduced to 17 factor variables.

#### General notes

All data analyses were conducted using versions 3.6.2 or 3.6.3 of the R program (R Core Team, 2018). Individual packages are cited in text when referenced. Before any analyses, all continuous variables were visually checked for outliers by plotting and comparing with similar curves, and any outliers were recoded to the largest/smallest value that fit the visual curve (cap method). Using this method only four observations were capped. All interactions were evaluated in the context of component main effects and all lower-level interactions, and continuous variables were centered.

#### Gender and UR status differences: logistic regression, multinomial logistic regression, and ANOVA

Gender and UR status differences on explanatory variables were investigated through three different procedures, depending on the nature of the explanatory (here dependent) variable. For all three sets of analyses, each analysis used the explanatory variables as dependent variables, and gender, UR status, and their interaction as the independent variables. Significant findings were followed up by examining differences either in means or slopes for subsamples defined by the moderating variable in question. False discovery rate (FDR) was controlled using the Benjamini and Hochberg (1995) procedure, which was applied to each analysis using the “BH” option on the mt.rawp2adjp function of the R Multtest package (Gentleman et al., 2005).

Gender and UR status differences on the four dichotomous explanatory variables were investigated using logistic regressions (using glm from the base package with family = “binomial” in R). Gender and UR status differences on the two multinomial explanatory variables were investigated using multinomial logistic regressions (using multinom from the nnet package in R; Venables and Ripley, 2002). Statistics for individual terms were computed by successively contrasting statistics from the full model to statistics from three submodels that each had a different term removed using the ANOVA test for model comparison. Finally, gender and UR status differences on the 33 continuous explanatory variables were investigated using ANOVA (using aov from the base package in R).

#### Differences in career interest ratings over time: repeated-measures MANOVAs

The four omnibus repeated-measures MANOVAs (one for each career type) were conducted using lmer from the lme4 package (Bates et al., 2015) in R (R Core Team, 2018). Each multivariate ANOVA (MANOVA) had all three interest ratings over time for a single career type as dependent variables, and time as the independent variable. Follow-ups were also conducted using lmer. Estimated marginal means and effect sizes were computed with emeans and eff_size from the emmeans package (Lenth, 2020). Follow-ups were conducted only for significant main effects or interactions from the omnibus MANOVAs. All degrees of freedom for follow-ups (Extended Data Fig. 5-1) were estimated using the Kenward–Roger method, and *p* values were adjusted to control for familywise error, using Tukey’s test.

#### Regressions investigating change in career interest during graduate school

FDR was controlled in all analyses using the Benjamini and Hochberg (1995) procedure, as above. In the first step, correlations between outcomes and explanatory variables were computed using corr.test from the psych package in R (Revelle, 2019). Outcome variables were the four T2 (end of PhD) ratings of interest in different careers with T1 (start of PhD) ratings of interest for the same career covaried out. Explanatory variables were limited to those that asked about graduate school and non-time-bound questions. Explanatory variables were carried over to third-step regressions if their correlations with outcome variables were significant to at least the *p* < 0.05 level, and they accounted for at least 2% of the variance in the outcome variables.

In the second step, interactions between graduate school-era explanatory variables, gender and UR status, predicting change in interest in the different careers over graduate school were tested using glm from the base package in R. Three-way interactions (gender by UR status by explanatory variable) for each of the explanatory variables predicting change in interest over graduate school were tested first. If the three-way interaction was not significant, then all two-way interactions predicting change in interest over graduate school were tested in a new equation that did not include the three-way interaction (to preserve shared variance). Interactions were carried over to third-step regressions if they were significant at least at the *p* < 0.05 level (no effect size requirements were used because there is no consensus effect size measure for moderation in the literature; Smithson and Shou, 2017).

In the third step, regressions were computed using lm from the base package in R. This step proceeded in two phases. In phase 1, regressions were computed including all explanatory variables and interactions brought forward from steps 1 and 2 (above). In phase 2, all variables and interactions that had semipartial correlation values of 0 in phase 1 were dropped, and new regression equations were computed. The effect size threshold for reporting results from these analyses was set to 1.0% unique variance captured, Cohen’s small effect for multiple regressions, because the loss of shared variance of each variable in the fitting of the multiple-regression model makes the amount of unique variance captured a stringent test.

Statistical assumptions for ordinary least-squares regressions were tested for all regressions in this step using the Breusch–Pagan test (for heteroskedasticity; bptest) and the Durbin–Watson test (for autocorrelated errors; dwtest) from the lmtest package in R (Zeileis and Hothorn, 2002). The means of the residuals for each regression were also checked to ensure that they were close to 0. Results showed that all the residual means were close to 0 and that none of the Durbin–Watson tests were significant. Many of the Breusch–Pagan tests were significant, however, and thus all regression coefficients, SEs, and *t* values were corrected for heteroskedasticity using vcovHC from the sandwich package in R (Zeileis, 2004).

#### Regressions predicting current interest

These analyses were conducted in an identical manner as the regression analyses above. Outcome variables were the four T3 (current) ratings of interest in the four different careers. The full set of explanatory variables were used as predictors.

## Results

### Sample demographics

Table 1 presents basic demographic information about the sample of 1479 PhD neuroscientists who responded to the survey. We solicited responses from all recent doctoral recipients (CY2008 or later) who were US citizens or permanent residents and had applied for NINDS funding or have been appointed to NINDS training (T32) or received research education grants (R25) between 2003 and 2017. Respondent information included gender (54% women, *n* = 793), UR status (16% UR, *n* = 233), and social identity (9% UR women, *n* = 133; 7% UR men, *n* = 100; 45% WR women, *n* = 660; and 40% WR men, *n* = 586). In addition, 41% of participants were in a postdoctoral position; 27% in research-focused academic positions; 10% in science-related, nonresearch positions; 9% in nonacademic research positions; 7% in teaching-focused academic positions; 4% in nonscience positions; and 2% unemployed. Also, 48% held a PhD in neuroscience, the rest were in biology or health-related fields; 251 PhD institutions were represented in 47 states, the District of Columbia, and Puerto Rico.

### Approach

After characterizing basic demographic information, we first investigated whether there were differences by social identity in factors likely to influence career interest, such as experiences in graduate school and postdoctoral training, personal characteristics, and objective measures. Next, we looked at whether there were changes in interest in the four career types (research-focused academic faculty positions; teaching-focused academic faculty positions; nonacademic research positions; and science-related, nonresearch positions) over time across the whole sample and by social identity. We then performed two different sets of follow-up analyses on career interests. The first set of analyses investigated which factors predicted changes in interest in the four career types over the course of graduate school. The second set of analyses investigated which factors predicted current interest in the four career types.

### Differences by gender and UR status in explanatory variables

First, we asked whether experiences in graduate school and postdoctoral training, personal characteristics, and objective measures differed by social identity in our sample. Significant findings were followed up by examining differences in either means or slopes for subsamples defined by whichever was significant of gender, UR status, or their interaction. Definitions for small, medium, and large effect sizes for this article are generally taken from Cohen (1988): mean differences use *d* [small (s) ≥ 0.2, medium (m) ≥ 0.5, large (l) ≥ 0.8], and correlation coefficients and individual regression coefficients use *r* (s ≥ 0.1, m ≥ 0.3, l ≥ 0.6). Finally, for odds ratios we used (rounded) Cohen’s cutoffs (s ≥ 1.5, m ≥ 2.5, l ≥ 4.5).

#### Gender

**Figure 1.**
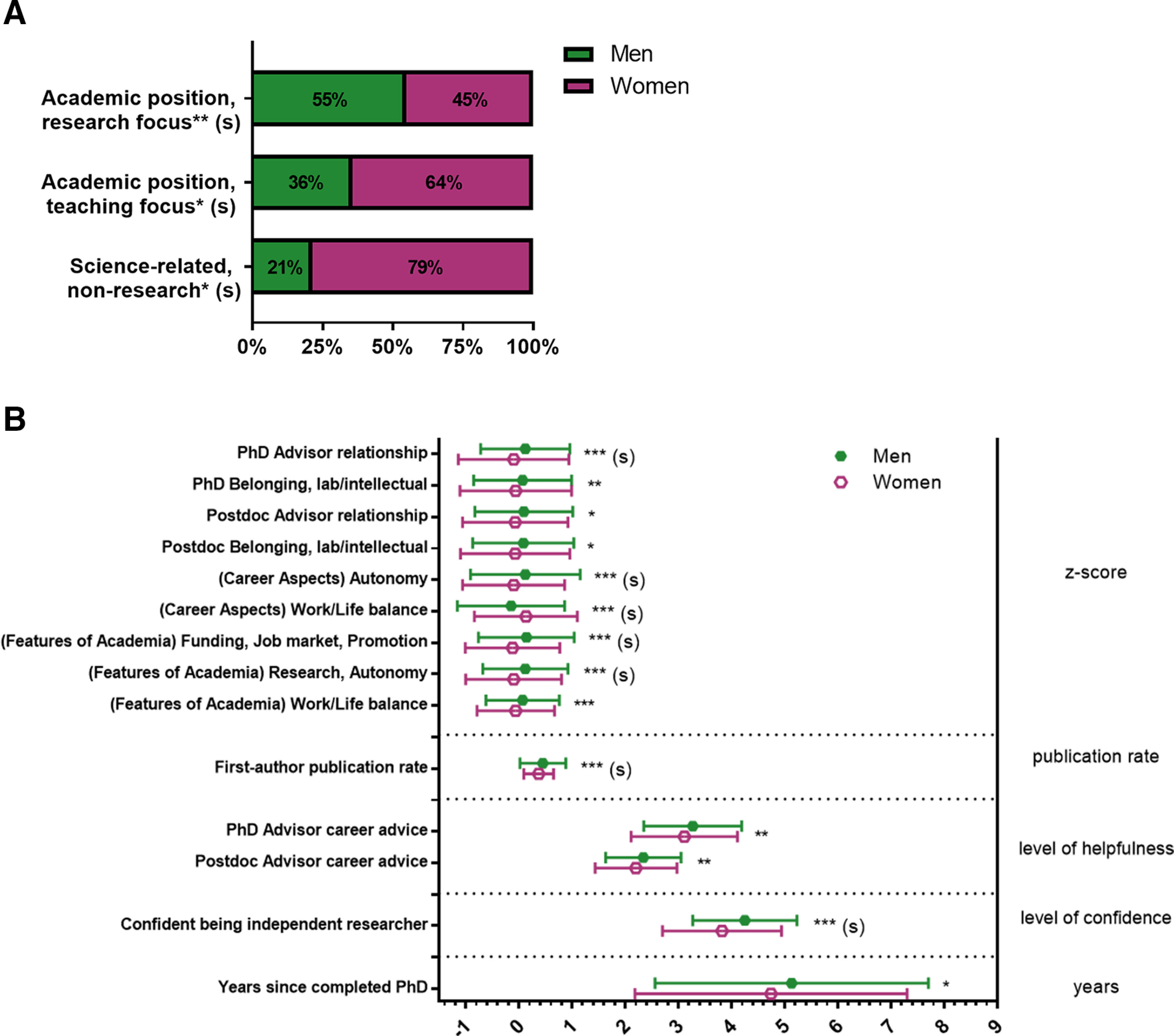
Gender differences among PhD neuroscientists. ***A***, Proportion of women and men in the sample by job sector of their current position. Significance levels from Chi-squared statistics (Extended Data Figure 1-1). ***B***, Mean responses to variables capturing experiences, personal characteristics, and objective measures. Significance levels from F statistics (ANOVA, Extended Data Figure 1-2) comparing the means for women and men for each variable were all significant at *p* < 0.05, at least. Responses on the X-axis were z-scores for the top variables; total publications/years of research for publication rate; level of helpfulness (1–4, 4 being very helpful) for the career advice variables; level of confidence (1–5, 5 being most confident) for confidence in being an independent researcher; and years for years since completed PhD. Effect sizes are labeled when they reach at least “small” size. (s) = small effect size, *p < 0.05, **p < 0.01, ***p < 0.001.

The follow-up analyses revealed several differences by gender in current position (Fig. 1*A*, Extended Data Fig. 1-1). A higher proportion of men than women were in research-focused academic faculty positions. Conversely, a higher proportion of women than men were in teaching-focused academic faculty positions and science-related, nonresearch positions.

10.1523/ENEURO.0163-21.2021.f1-1 Extended Data Figure 1-1 Contingency table for gender by current position association. Follow-up analyses performed on significant findings in gender by current position. Effect size: (-) = negligible effect size, (s) = small effect size. * = p < 0.05, ** = p < 0.01. Download Figure 1-1, DOC file.

**Figure 2.**
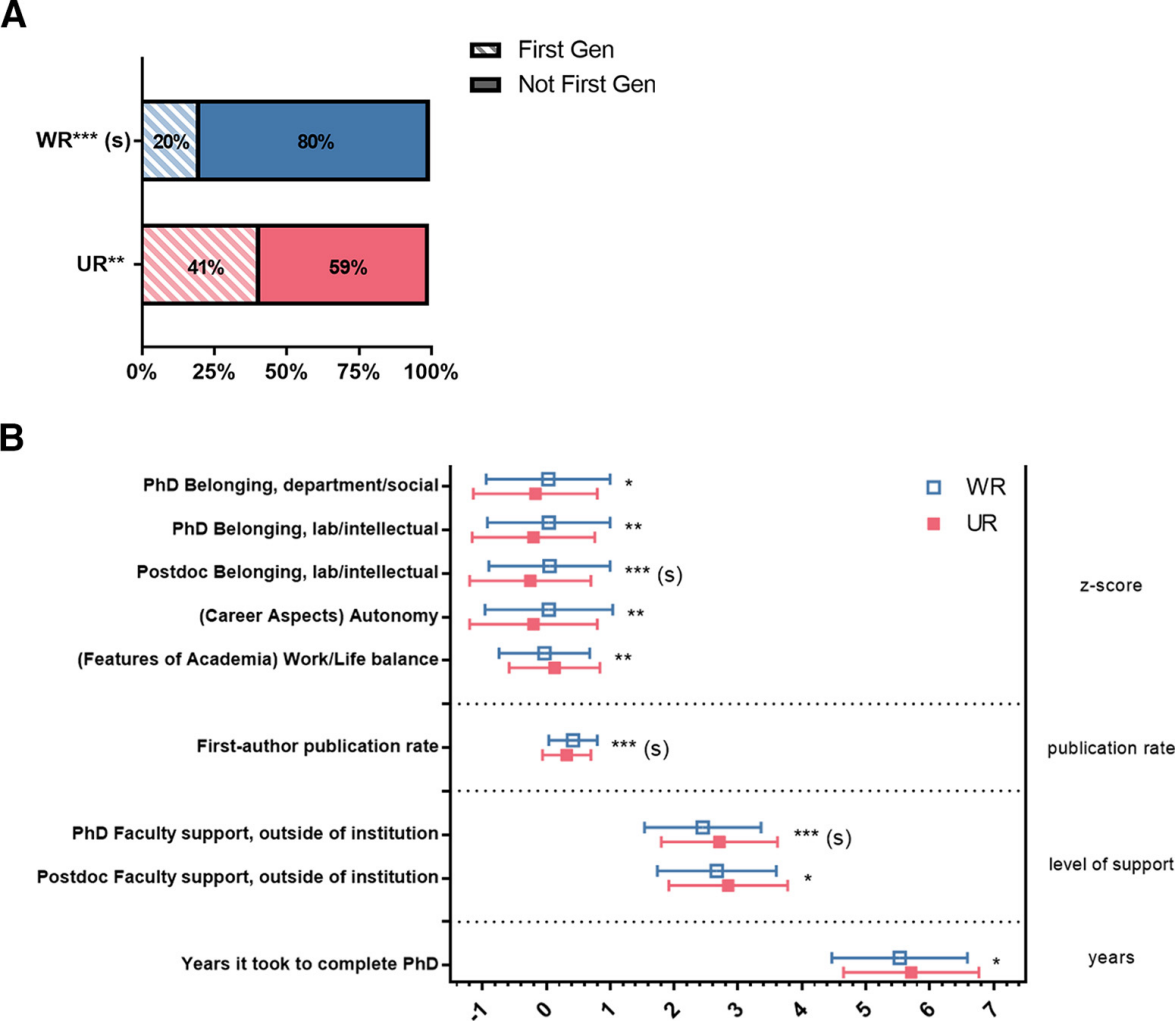
Differences between WR and UR respondents. ***A***, WR respondents were less likely to be first generation college students than UR respondents. Proportion of respondents who were the first person or in the first generation in their family to graduate from a 4-year college by UR status. Significance levels from Chi-squared statistics. (Extended Data Figure 2-1). ***B***, Mean responses to variables capturing experiences, personal characteristics, and objective measures. Significance levels from F statistics (ANOVA, Extended Data Figure 2-2) comparing the means for UR respondents and WR respondents for each variable were all significant at *p* < 0.05. Responses on the X-axis were z-scores for the top variables; total publications/years of research for publication rate; level of helpfulness (1–4, 4 being very helpful) for the outside faculty support variable; and years for years it took to complete PhD. Effect sizes are labeled when they reach at least “small” size. (s) = small effect size; *p < 0.05, **p < 0.01, ***p < 0.001.

10.1523/ENEURO.0163-21.2021.f1-2 Extended Data Figure 1-2 Means for continuous explanatory variables split by gender. Follow-up analyses performed on significant findings in explanatory variables by examining differences in means for subsamples split by gender. N = number in group, M = mean, n = number in subgroup, SD = standard deviation. Effect size: (-) = negligible effect size, (s) = small effect size. * = p < 0.05, ** = p < 0.01, *** = p < 0.001. Download Figure 1-2, DOC file.

We also found differences in experiences and personal characteristics between men and women (Fig. 1*B*, Extended Data Fig. 1-2). Women’s assessments of their relationships with their PhD program advisors were significantly lower than those from men, as reflected by the negative *z* scores. Women also report a significantly lower publication rate than men. In addition, women’s current ratings of their confidence in their potential to be independent researchers were significantly lower than those of men. For the factors assessing the importance of different aspects of careers, women rated the “autonomy” factor less important and the “work/life balance” factor more important than men. Finally, for the factors assessing whether different “features of academia” increased or decreased interest in academia, women reported that the “funding/job market/promotion” factor and the “research/autonomy” factor decreased their interest in academia more than men.

#### UR status

WR and UR respondents also differed on several variables. We found that WR respondents were far less likely than UR respondents to have been the first person or in the first generation of their family to graduate from a 4 year college or university (Fig. 2*A*, Extended Data Fig. 2-1). We also found differences between UR and WR respondents in experiences and personal characteristics (Fig. 2*B*, Extended Data Fig. 2-2). UR respondents reported feeling more support from faculty outside their institutions during their PhD programs than WR respondents. Conversely, UR respondents had lower scores on the factor that captured feelings of belonging intellectually/socially to their postdoctoral research group than WR respondents. UR respondents also reported lower publication rates than WR respondents. For the factors assessing the importance of different aspects of careers, UR respondents reported lower importance of the autonomy factor than WR respondents. Finally, for the factors assessing the influence of different features of academia, UR respondents reported that the work/life balance factor increased their interest in academia more than WR respondents.

10.1523/ENEURO.0163-21.2021.f2-1 Extended Data Figure 2-1 Contingency table for UR Status by Family College Graduation History association. Follow-up analyses performed on significant findings in UR Status by Family College Graduation History. UR = underrepresented. Effect size: (-) = negligible effect size, (s) = small effect size. ** = p < 0.01, *** = p < 0.001. Download Figure 2-1, DOC file.

10.1523/ENEURO.0163-21.2021.f2-2 Extended Data Figure 2-2 Means for continuous explanatory variables split by UR Status. Follow-up analyses performed on significant findings in explanatory variables by examining differences in means for subsamples split by UR Status. UR = underrepresented, WR = well represented. N = number in group, M = mean, n = number in subgroup, SD = standard deviation. Effect size: (-) = negligible effect size, (s) = small effect size. * = p < 0.05, ** = p < 0.01, *** = p < 0.001. Download Figure 2-2, DOC file.

#### Gender and UR status

**Figure 3.**
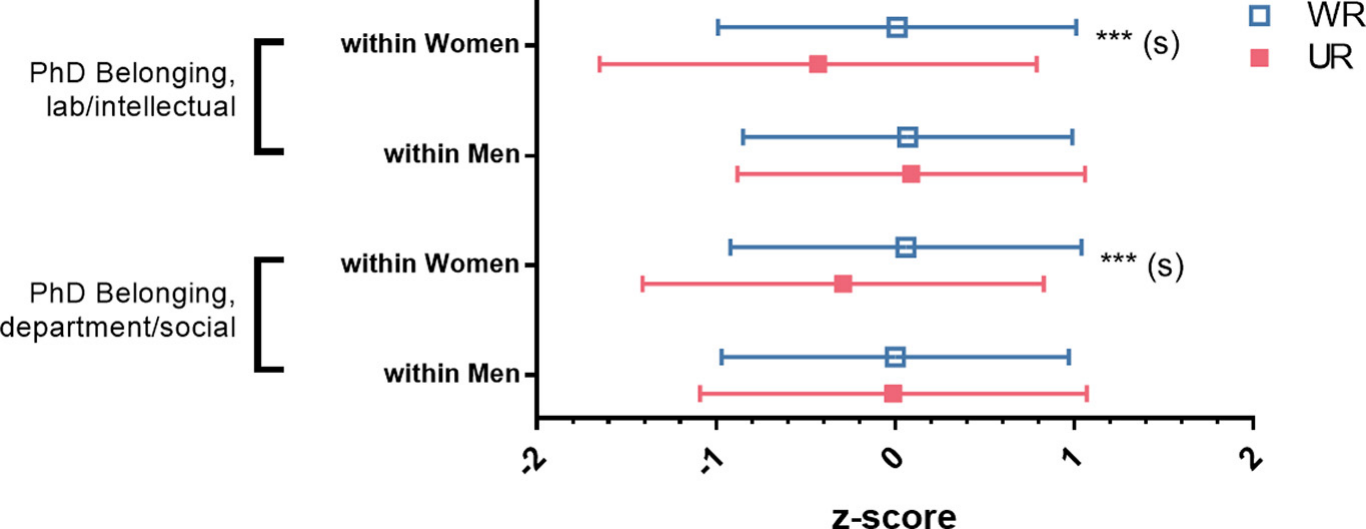
UR women feel a lower sense of belonging than WR women, with no difference for men. Mean responses split by gender and UR status on their feelings of belonging to their PhD lab/research group or department (reduced to 2 single factors with factor analysis). Significance levels from F statistics (ANOVA, Extended Data Figure 3-1). Effect sizes are labeled when they reach at least “small” size. (s) = small effect size; *p < 0.05, **p < 0.01, ***p < 0.001.

At the intersection of social identity (gender and UR status), we found a two-way interaction of gender and UR status for feelings of belonging in both the PhD research group and PhD department (Fig. 3, Extended Data Fig. 3-1). Although there was no difference between WR and UR men, UR women reported lower feelings of belonging than WR women on both the factor that captured feelings of intellectual belonging to their PhD laboratory/research group and the factor that captured feelings of social belonging to their PhD department.

10.1523/ENEURO.0163-21.2021.f3-1 Extended Data Figure 3-1 Follow-ups for continuous explanatory variables that had significant interactions of Gender by UR Status. Follow-up analyses performed on significant findings in explanatory variables by examining differences in means for subsamples by interaction between gender and UR Status. UR = underrepresented, WR = well represented. N = number in group, M = mean, n = number in subgroup, SD = standard deviation. Effect size: (-) = negligible effect size, (s) = small effect size. * = p < 0.05, *** = p < 0.001. Download Figure 3-1, DOC file.

### Changes in career interest over time

**Figure 4.**
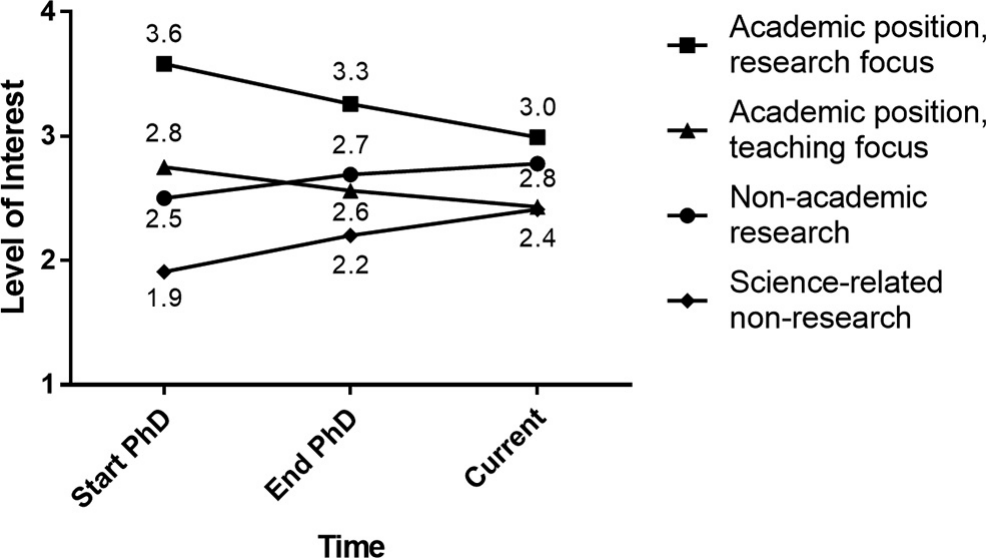
Change in career interest ratings over time among PhD neuroscientists. Mean responses of 1,479 PhD neuroscientists who were asked to rate their level of interest in four different career paths at three times: Start of PhD (T1), End of PhD (T2), and Current (T3), on a 4-point scale (where 1 represents “no interest” and 4 represents “strong interest”). Repeated measures MANOVAs found T1 v T2 was significant at *p* < 0.001 for all careers; T2 v T3 was significant at *p* < 0.001 for Academic Faculty, research focus and Science-related non-research, and *p* < 0.01 for academic faculty, teaching focus and non-academic research (Extended Data Figure 4-1).

We were interested in whether there were changes in the four career interest ratings over time across the entire sample. Using repeated-measures MANOVAs, we found significant main effects for time for all four career types (Fig. 4, Extended Data Fig. 4-1): decreases over time for interest in research-focused academic faculty positions and teaching-focused academic faculty positions, and increases over time for nonacademic research positions and science-related, nonresearch positions. This reflects trends that are similar to those in other studies: interest in academia, both research- and teaching-focused positions, goes down over time, while interest in nonacademic careers goes up over time (Golde and Dore, 2001; Fuhrmann et al., 2011; Goulden et al., 2011; Sauermann and Roach, 2012; Gibbs et al., 2014, 2015; Roach and Sauermann, 2017; but see Wood et al., 2020).

10.1523/ENEURO.0163-21.2021.f4-1 Extended Data Figure 4-1 Omnibus MANOVA means for Change in Career Interest Ratings Over Time. Results from four separate omnibus repeated measures ANOVAs to ascertain whether there were differences in the 4 career interest ratings over time (within-subjects ordinal independent variable). SD = standard deviation. ** = p < 0.01, *** = p < 0.001. Download Figure 4-1, DOC file.

**Figure 5.**
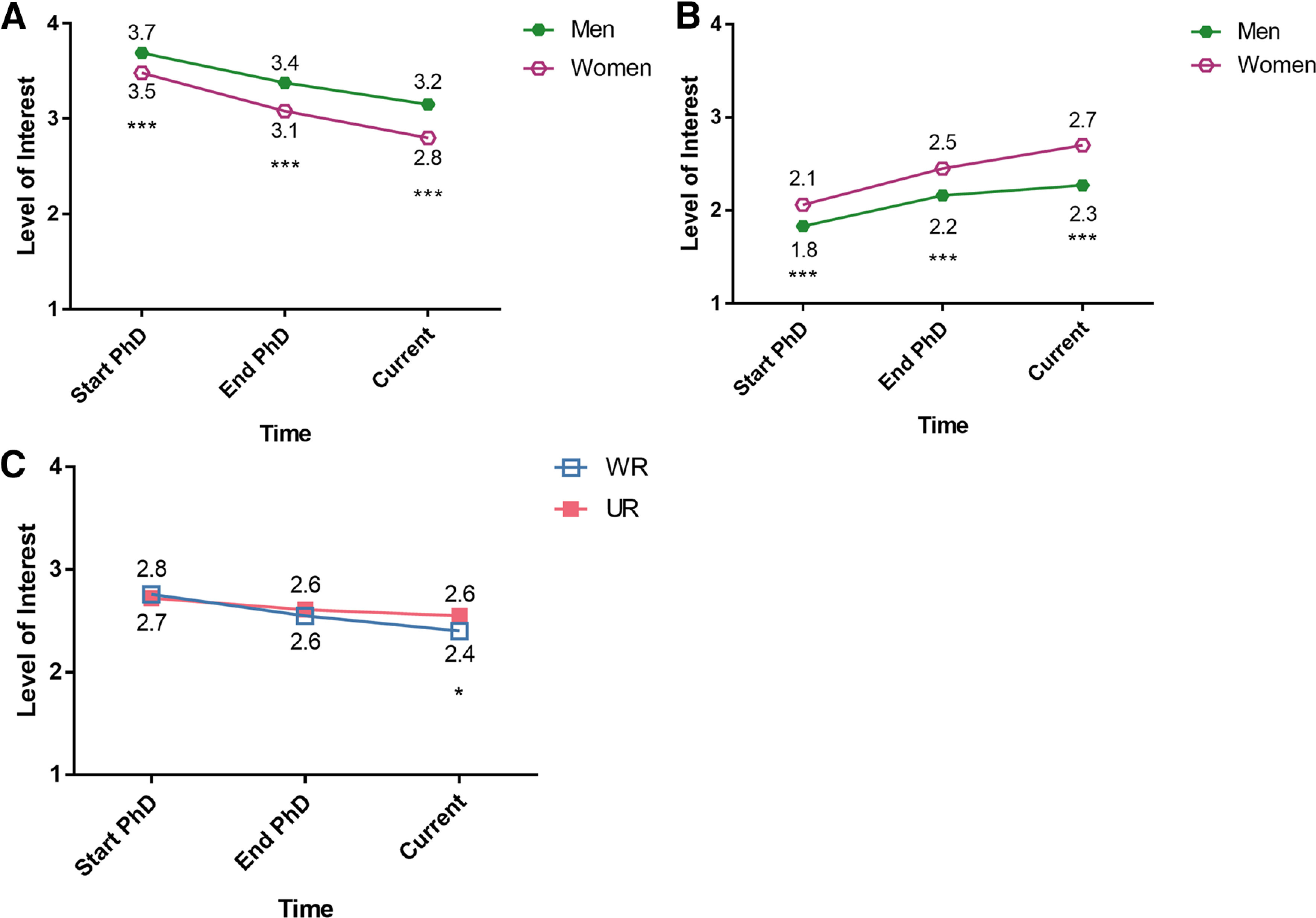
Follow-up analyses for significant interactions with Gender and UR status. ***A***, Women less interested in academic research positions than men at PhD start and over time, and interest decreased at a higher rate. Mean responses split by gender on level of interest in research-focused academic faculty positions at Start of PhD (T1), End of PhD (T2),and Current (T3) on a 4-point scale (where 1 represents “no interest” and 4 represents “strong interest”). Omnibus repeated measures MANOVA found an interaction between time and gender. ***B***, Women more interested in science-related, non-research positions than men at PhD start and over time, and their interest increased at a higher rate. Mean responses split by gender on level of interest in science-related, non-research positions at Start of PhD (T1), End of PhD (T2), and Current (T3) on a 4-point scale (where 1 represents “no interest” and 4 represents “strong interest”). Omnibus repeated measures MANOVA found an interaction between time and gender. ***C***, WR respondents less interested in academic teaching positions following PhD completion. Mean responses split by UR status on level of interest in teaching-focused academic faculty positions at Start of PhD (T1), End of PhD (T2), and Current (T3) on a 4-point scale (where 1 represents "no interest" and 4 represents “strong interest”). Omnibus repeated measures MANOVA found an interaction between time and UR status. Significance levels from follow-up ANOVAs (Extended Data Figure 5-1). *p < 0.05, ***p < 0.001.

To determine whether there were differences in trajectory of interest over time by social identity, follow-up analyses were conducted for significant interactions with gender and UR status (Extended Data Fig. 5-1). For interest in research-focused academic faculty positions, the “time by gender” interaction indicated that women’s interest in research-focused academic faculty positions was lower at the start of training and decreased over time at a higher rate than men’s interest (Fig. 5*A*). For interest in science-related, nonresearch positions, the time by gender interaction indicated that women’s interest in science-related, nonresearch positions increased over time at a higher rate than men’s interest (Fig. 5*B*). Finally, for interest in teaching-focused academic faculty positions, the “time by UR status” interaction indicated that well represented participants’ interest in teaching-focused academic faculty positions decreased over time at a higher rate than UR participants’ interest (Fig. 5*C*).

10.1523/ENEURO.0163-21.2021.f5-1 Extended Data Figure 5-1 Follow-ups for significant interactions in Omnibus MANOVA Change in Career Interest Ratings Over Time. Investigating whether Gender or UR Status (between-subjects independent variables) were moderators of differences in the 4 career interest ratings over time. UR = underrepresented, WR = well represented. SD = standard deviation. ** = p < 0.01, *** = p < 0.001. Download Figure 5-1, DOC file.

### Predicting change in interest

Next, we were interested in determining which, if any, factors predicted changes in career interest over the course of a PhD program. For this analysis, we chose predictor variables that captured issues that were contemporaneous with participants’ time in PhD training. Descriptions of the procedures can be found in the Materials and Methods section, and preliminary steps and results for construction of the regression are reported in Extended Data Tables 2-1 and 2-2.

**Table 2:** Regressions Predicting Career Interest Ratings at T2 (End of PhD) from Graduate School Era Explanatory Variables

Independent variables (graduate school era explanatory)	Dependent variables T2 (end of PhD) career interest ratings											
	Academic faculty/research			Academic faculty/teaching			Nonacademic research			Science/nonresearch		
	Unstd. Coeff.	Sig.	Effect size	Unstd. Coeff.	Sig.	Effect size	Unstd. Coeff.	Sig.	Effect size	Unstd. Coeff.	Sig.	Effect size
Variables in the equation												
(Intercept)	2.682	***		2.359	***		2.662	***		2.442	***	
T1 (start of PhD) interest rating	0.597	***	M (0.174)	0.696	***	L (0.421)	0.536	***	M (0.148)	0.766	***	L (0.323)
T1 interest rating-BY-gender							0.132	**				
T1 interest rating-BY-UR status										−0.135	*	
PhD belonging, laboratory	0.091	**										
PhD belonging, laboratory-BY-gender												
PhD belonging, laboratory-BY-UR status												
PhD belonging, lab-BY-gender-BY-UR status												
PhD advisor career advice	0.235	***	S (0.045)	0.113	***					−0.124	***	S (0.014)
PhD advisor career advice-BY-UR status				−0.115								
PhD faculty support, at institution				−0.010								
PhD faculty support, at institution-BY-gender				0.022								
PhD faculty support, at institution-BY-UR status				0.089								
PhD faculty support, at institution-BY-gender-BY-UR status				−0.311	*							
PhD faculty support, outside institution	0.008											
PhD faculty support, outside institution-BY-gender	0.029											
PhD faculty support, outside institution-BY-UR status	0.124											
PhD faculty support, outside institution-BY-gender-BY-UR	−0.304	*										
Gender	0.097			−0.121			0.049					
UR	−0.266			0.085						0.236	***	
Gender-BY- UR	0.446			0.792	*							
Model statistics													
*R*^2^	0.309			0.436			0.339			0.419		
Adjusted *R*^2^	0.304			0.432			0.337			0.417		
*F*	65.68			113.29			251.81			265.55		
df	11			11			4			5		
df.residual	1468			1468			1475			1474		
*p*	0.0000			0.0000			0.0000			0.0000		
Non-T1-interest variance	0.14			0.01			0.19			0.10		

Results of regressions for each of the four career interest T2 ratings (end of PhD) on T1 (start of PhD) interest rating, significantly correlated variables (Extended Data Table 2-1), and significant predictor interactions (Extended Data Table 2-2). Unstd. Coeff. = Unstandardized coefficient; sig. = significance. Effect sizes are labeled when they reach at least “small” size. (S) = small effect size, (M) = medium effect size, (L) = large effect size. *p < 0.05; **p < 0.01; ***p < 0.001.

10.1523/ENEURO.0163-21.2021.t2-1 Extended Data Table 2-1 Correlations for T1->T2 Regressions. Career interest at T2 was regressed on T1 ratings, and correlations with the independent variables were computed with residuals from those procedures (adjusted outcomes). T1 = Time 1 (Start of PhD), T2 = Time 2 (End of PhD). * = p < 0.05, ** = p < 0.01, *** = p < 0.001. Shaded: > 2% variance. Download Table 2-1, DOC file.

10.1523/ENEURO.0163-21.2021.t2-2 Extended Data Table 2-2 Interaction Tests for T1->T2 Regressions. Abbreviated results for investigation of interactions between the graduate school era explanatory variables and gender and UR status. IV = independent variable, T1 = Time 1 (Start of PhD), T2 = Time 2 (End of PhD). * = p < 0.05, ** = p < 0.01, *** = p < 0.001. Download Table 2-2, DOC file.

The final step in this analysis was to regress each of the four career interest ratings at the end of graduate school (T2) on the following: (1) the interest rating for the same career at the start of graduate school (T1), (2) the variables that had significant correlations with it in the correlation step (Extended Data Table 2-1), and (3) the interactions that were significant predictors of it in the interaction test step (Extended Data Table 2-2). Because the T1 interest rating is in the equation simultaneously with the other variables, it is interpreted as predicting change in interest during graduate school. The results of these analyses are presented in Table 2. We discuss the regression for each career type in the following subsections. Discussion of each regression includes only coefficients/variables that were significant and had at least a small effect size. Significant main effects in the context of interactions are not discussed, and significant lower-level interactions in the context of higher-level interactions are not discussed fully, because their meaning is difficult to determine in that context.

#### Factors that predict changes in interest in research-focused academic positions

**Figure 6.**
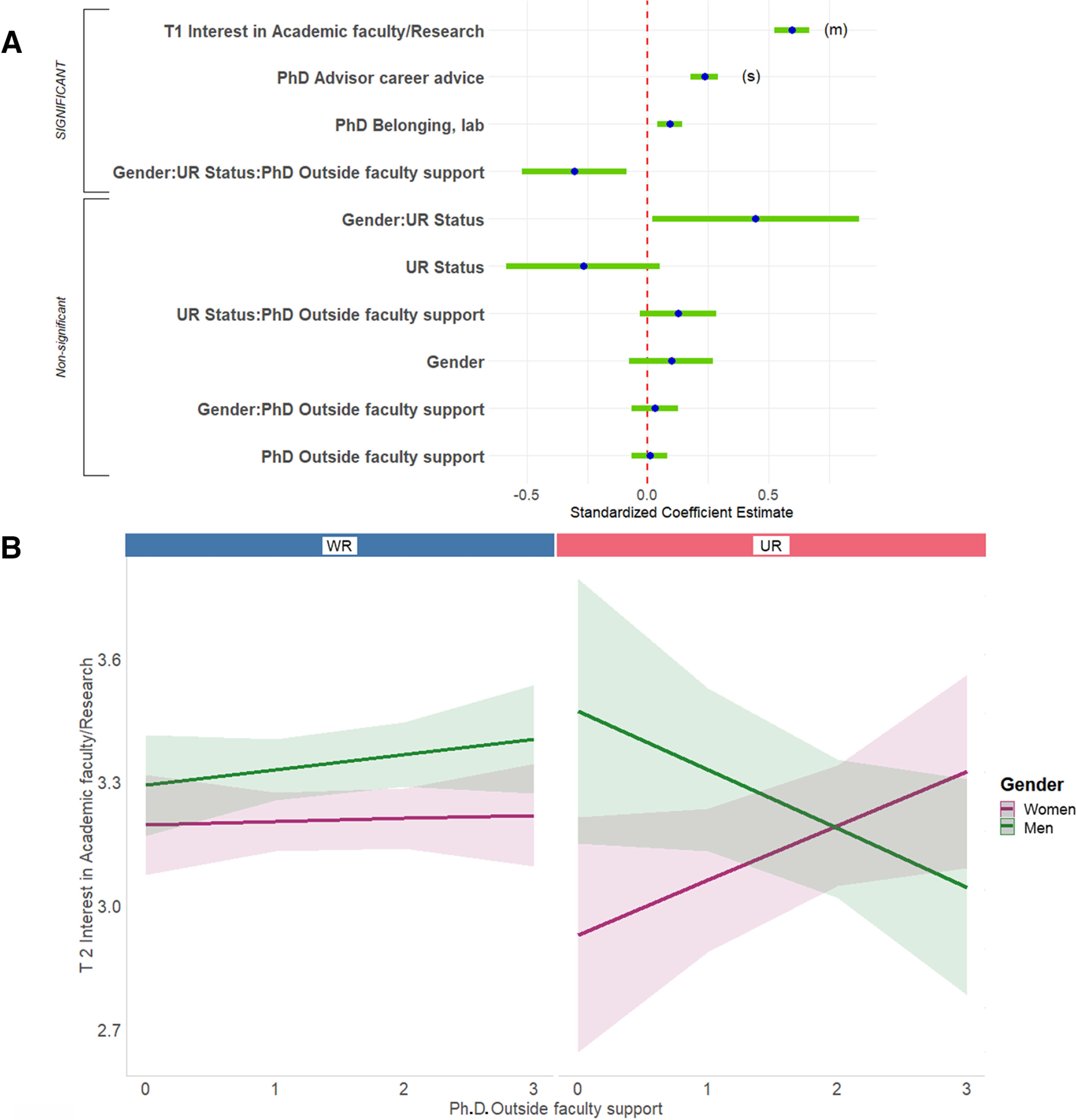
Predicting end of PhD interest in research-focused academic faculty positions. ***A***, Standardized regression coefficients and error bars for linear regression predicting interest at the end of PhD training (T2) in research-focused academic faculty positions. Dependent variables were level of interest at the end of PhD training on a 4-point scale (where 1 represents “no interest” and 4 represents “strong interest”). Independent variables captured level of interest at the start of PhD training (T1), experiences during PhD training, personal characteristics, objective measures, and interactions with gender and UR status. The entire equation was significant at *p* < 0.001 and captured 30.4% of the variance (adjusted; Table 2). Effect sizes are labeled when they reach at least “small” size. (s) = small effect size, (m) = medium effect size. ***B***, Outside faculty support during PhD associated with increased interest in research-focused academic faculty positions for UR women, but decreased interest in UR men, no gender difference in WR respondents. Regression lines predicting interest at the end of PhD training (T2) in research-focused academic faculty positions from level of outside faculty support during PhD training, split by gender and UR status. Dependent variable was level of interest at the end of PhD training on a 4-point scale (where 1 represents “no interest” and 4 represents “strong interest”). Independent variable was level of helpfulness of outside faculty (0-3, 3 being very helpful). Interaction was significant at *p* < 0.05 (Table 2; Extended Data Figure 6-1).

First, we predicted change in respondents’ ratings of interest in research-focused academic faculty positions from the start of graduate school (T1) to the end of graduate school (T2). The full regression equation (Fig. 6*A*, all predictors) was itself significant, accounting for 30.4% of the variance in interest in research academia at T2 (adjusted *R*^2^; Table 2, Extended Data Fig. 6-1). Note that interest at the start of graduate school predicted a modest 17.4% of the variance. Helpfulness of career advice from PhD advisor (4.5% variance) was the only other significant predictor with sufficient effect size to report. Higher ratings of the helpfulness of career advice from PhD advisors was also related to greater interest in academic research careers during graduate school. Although other single variables were significant predictors, their effect sizes did not meet the threshold for reporting.

10.1523/ENEURO.0163-21.2021.f6-1 Extended Data Figure 6-1 Follow-ups for significant interactions in regressions predicting T2 interest. Follow-up results for significant interactions in the final regressions reported in Table 2. UR = underrepresented, WR = well represented. * = p < 0.05, ** = p < 0.01, *** = p < 0.001. Download Figure 6-1, DOC file.

The regression analysis identified a significant three-way interaction among gender, UR status, and level of support from faculty outside respondents’ institutions during their PhD program (hereafter, called “PhD outside faculty support”). The interaction, and all other three-way interactions in this article, was followed up by testing gender differences in prediction of the dependent variable by the explanatory variable within levels of UR status. Although higher support from outside faculty was related to an increase in interest in academic research over the course of graduate school for UR women, it was related to a decrease in interest for UR men (Fig. 6*B*; there was no relation for WRs).

#### Factors that predict changes in interest in teaching-focused academic positions

**Figure 7.**
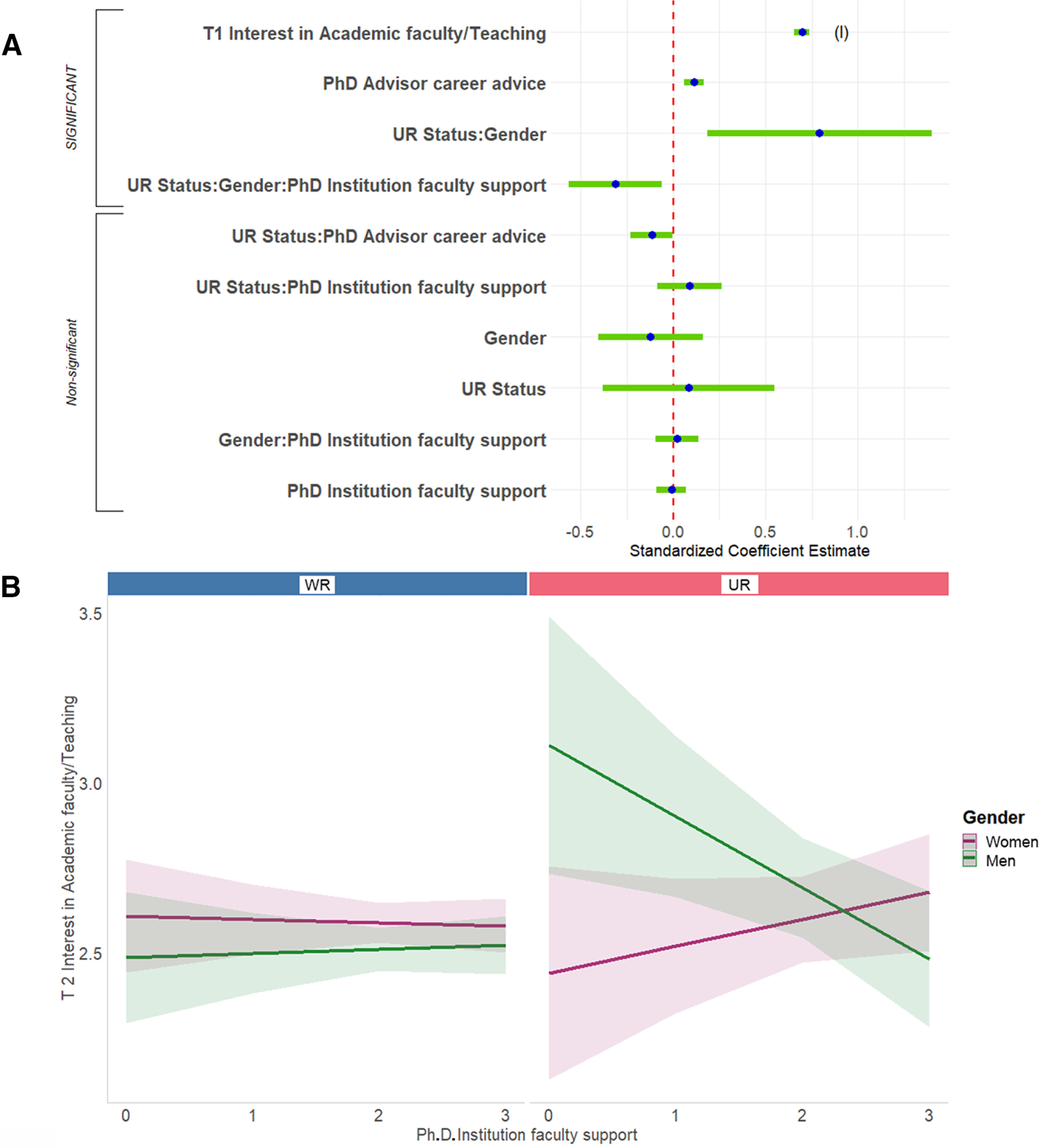
Predicting T2 interest in teaching-focused academic faculty positions. ***A***, Standardized regression coefficients and error bars for linear regression predicting interest at the end of PhD training (T2) in teaching-focused academic faculty positions. Dependent variables were level of interest at the end of PhD training on a 4-point scale (where 1 represents “no interest” and 4 represents “strong interest”). Independent variables captured level of interest at the start of their PhD training (T1), experiences during PhD training, personal characteristics, objective measures, and interactions with gender and UR status. The entire equation was significant at *p* < 0.001 and captured 43.2% of the variance (adjusted; Table 2). Effect sizes are labeled when they reach at least “small” size. (l) = large effect size. ***B***, PhD institution faculty support was associated with increased interest in teaching-focused academic faculty positions for UR women, but was associated with decreased interest in UR men, no gender difference in WR respondents. Regression lines predicting interest at the end of PhD training (T2) in teaching-focused academic faculty positions from level of institution faculty support during PhD training, split by gender and UR status. Dependent variable was level of interest at the end of their PhD training (T2) on a 4-point scale (where 1 represents “no interest” and 4 represents “strong interest”). Independent variable was level of helpfulness of PhD institution faculty (0–3, 3 being very helpful). Interaction was significant at *p* < 0.05 (Table 2; Extended Data Figure 6-1).

Second, we predicted change in respondents’ ratings of interest in teaching-focused academic faculty positions. The full regression equation was significant, accounting for 43.2% of the variance in T2 interest (Fig. 7*A*, Table 2, adjusted *R*^2^). Interest in academic teaching at the start of graduate school predicted interest in academic teaching at the end of graduate school to a high degree (42.1% of the variance). Again, although other single variables were significant predictors, their effect sizes did not meet the threshold for reporting.

A three-way, interaction very similar to the interaction discussed in the last section, among gender, UR status, and level of support from faculty at respondents’ institutions during their PhD program (hereafter called “PhD institution faculty support”) was significant. Although there was no relation for WR respondents, PhD institution faculty support was associated with increased interest in teaching-focused academic faculty positions for UR women, but greatly decreased interest in UR men (Fig. 7*B*).

#### Factors that predict changes in interest in nonacademic research positions

**Figure 8.**
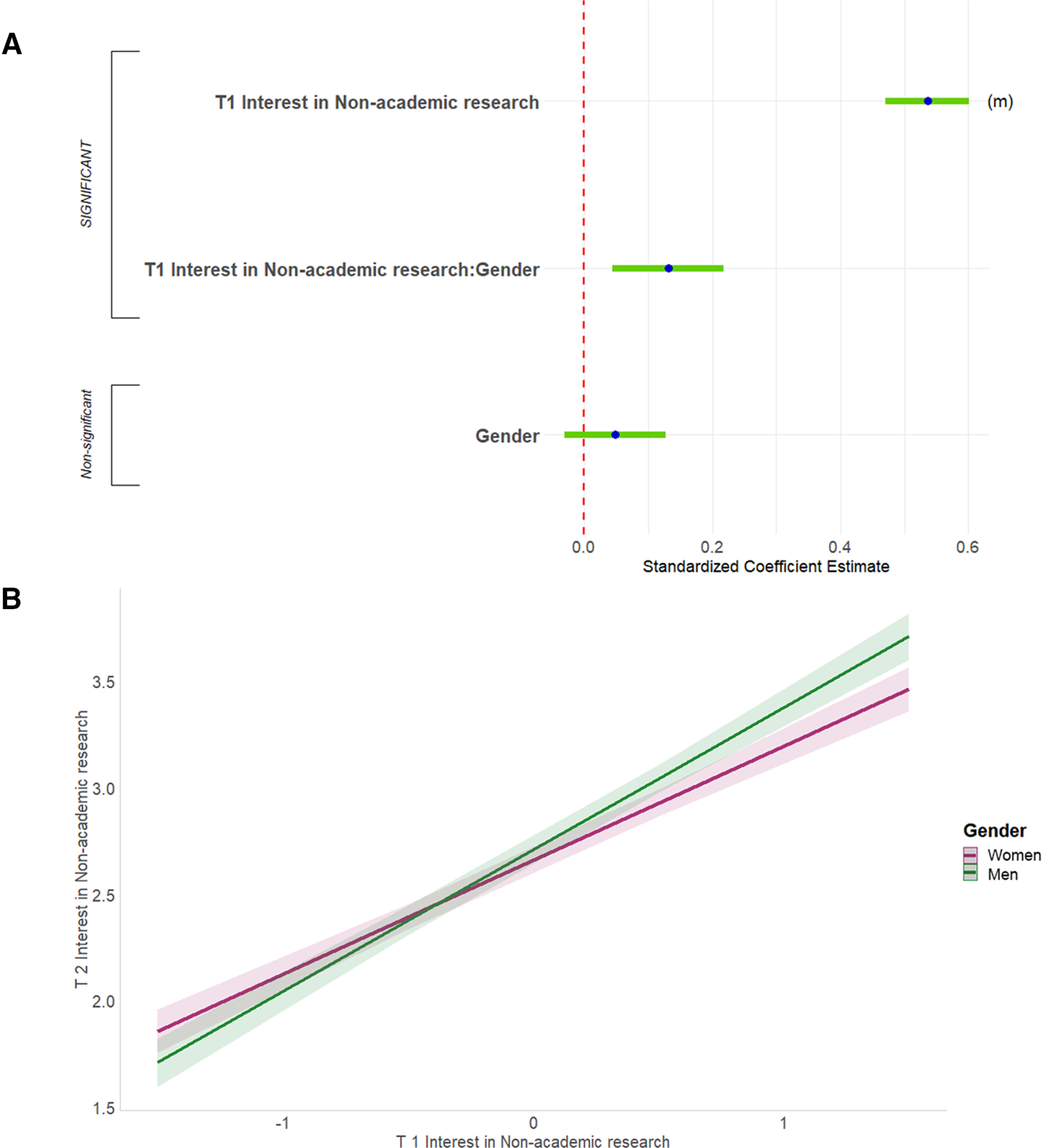
Predicting T2 interest in non-academic research positions. ***A***, Standardized regression coefficients and error bars for linear regression predicting interest at the end of PhD training (T2) in research/non-academic positions. Dependent variable was level of interest at the end of their PhD training (T2) on a 4-point scale (where 1 represents “no interest” and 4 represents “strong interest”). Independent variables captured level of interest at the start of their PhD training (T1), experiences during PhD training, personal characteristics, objective measures, and interactions with gender and UR status. The entire equation was significant at *p* < 0.001 and captured 33.7% of the variance (adjusted; Table 2). Effect sizes are labeled when they reach at least “small” size. (m) = medium effect size. ***B***, Graph shows regression lines predicting interest at the end of PhD training (T2) in research/non-academic positions from T1 interest, split by gender. Dependent variable from 1,479 PhD neuroscientists who were asked to rate their level of interest at the end of their PhD training (T2) on a 4-point scale (where 1 represents “no interest” and 4 represents “strong interest”). Independent variable was interest at T1, centered for interaction (-2 to 2). Interaction was significant at *p* < 0.01 (Table 2; Extended Data Figure 6-1).

Third, we predicted change in respondents’ ratings of interest in nonacademic research positions. The full regression equation was significant, accounting for 33.7% of the variance (Fig. 8*A*, Table 2, adjusted *R*^2^). Interest in nonacademic research positions at the start of graduate school predicted interest in nonacademic research positions at the end of graduate school to a moderate degree (14.8% of the variance). This finding was in the context, however, of a significant two-way interaction between gender and T1 rating of interest in nonacademic research positions. The association between interest at the start of graduate school and interest at the end of graduate school was somewhat stronger for men than it was for women (Fig. 8*B*).

#### Factors that predict changes in interest in science-related, nonresearch positions

**Figure 9.**
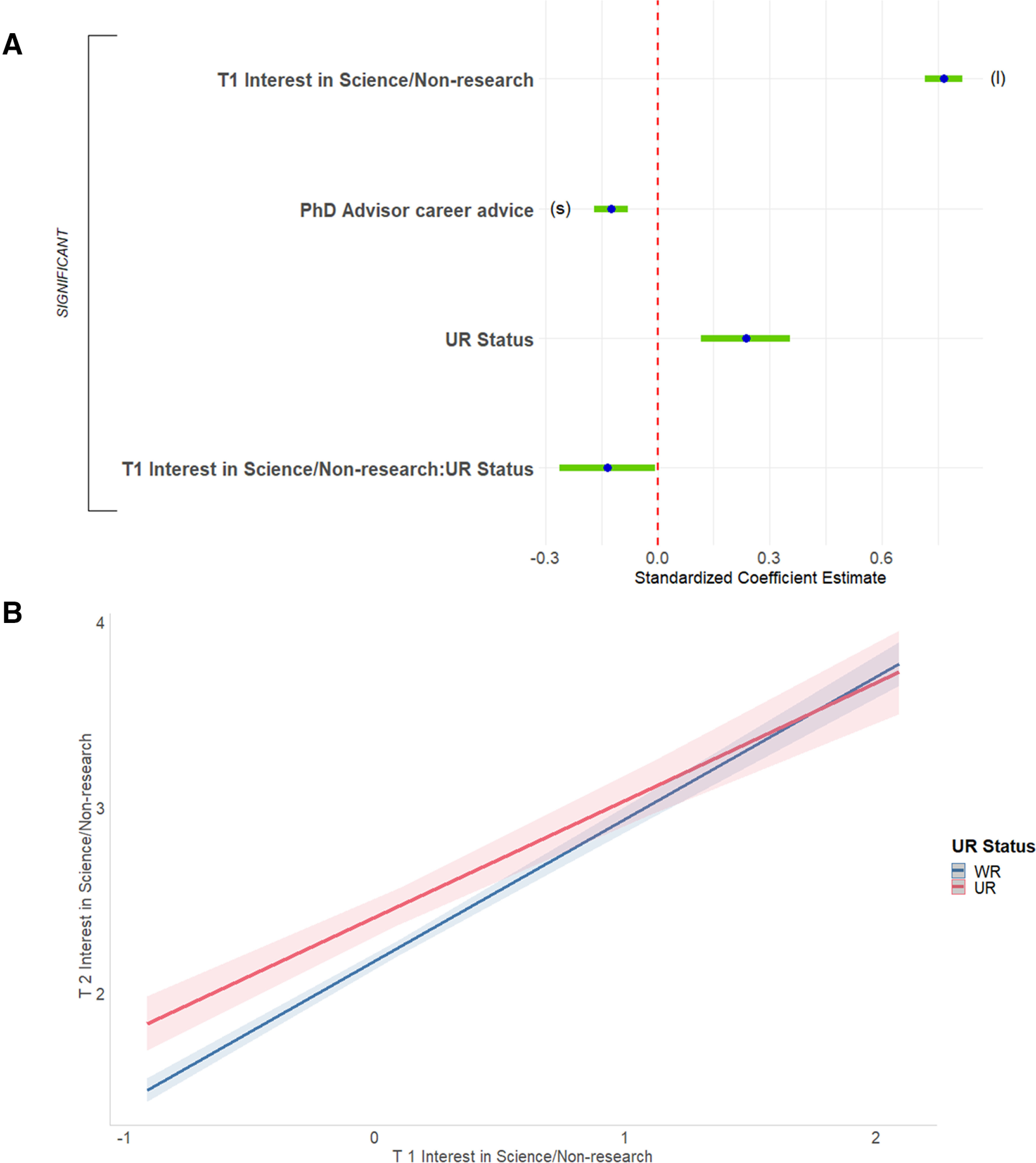
Predicting T2 interest in science-related, non-research positions. ***A***, Standardized regression coefficients and error bars for linear regression predicting interest at the end of PhD training (T2) in science-related, non-research positions. Dependent variable was level of interest at the end of their PhD training (T2) on a 4-point scale (where 1 represents “no interest” and 4 represents “strong interest”). Independent variables captured level of interest at the start of their PhD training (T1), experiences during PhD training, personal characteristics, objective measures, and interactions with gender and UR status. The entire equation was significant at *p* < 0.001 and captured 41.7% of the variance (adjusted; Table 2). Effect sizes are labeled when they reach at least “small” size. (l) = large effect size. ***B***, T1 interest in science-related, non-research positions was a stronger predictor for T2 interest for WR than UR respondents. Regression lines predicting interest at the end of PhD training (T2) in science-related, non-research positions from interest at the start of PhD training (T1), split by UR status. Dependent variable was level of interest at the end of PhD training on a 4-point scale (where 1 represents “no interest” and 4 represents “strong interest”). Independent variable was interest at T1, centered for interaction (-2 to 2). Interaction was significant at *p* < 0.05 (Table 2; Extended Data Figure 6-1).

Finally, we predicted change in respondents’ ratings of interest in science-related, nonresearch positions. The full regression equation was significant, accounting for 41.7% of the variance (Fig. 9*A*, Table 2, adjusted *R*^2^). Interest in science-related, nonresearch positions at the start of graduate school predicted interest in science/nonresearch at the end of graduate school to a high degree (32.3% of the variance; this finding was in the context, however, of a significant two-way interaction, below). Helpfulness of career advice from PhD advisor was also significant. This finding was in the context, however, of a significant two-way interaction with a moderate effect size. Lower ratings of the helpfulness of career advice from PhD advisors was related to becoming more interested in science-related, nonresearch positions across graduate school (1.4% of the variance).

A single two-way interaction, between UR status and T1 rating of interest in science-related, nonresearch positions, was significant for this equation. The association between T1 interest and T2 interest was somewhat stronger for WRs than it was for URs (Fig. 9*B*).

### Predicting current interest

We were also interested in which factors predicted current interest in different careers. For this analysis, we chose predictor variables that captured issues that were either current, or indicative of participants’ time in postdoctoral positions. Descriptions of the procedures can be found in the Materials and Methods section, and preliminary steps and results for construction of the regression are reported in Extended Data Tables 3-1 and 3-2.

The final step in this set of analyses was to conduct regression analyses to predict current interest in the different careers. Each equation was constructed by regressing one of the four career interest ratings at T3 (current) on the following: (1) the variables that had significant correlations with it in the first step (Extended Data Table 3-1), and (2) the interactions that were significant predictors of it in the second step (Extended Data Table 3-2). The results of these analyses are presented in Table 3. We discuss the regression for each career type in the following subsections.

**Table 3:** Regressions Predicting Career Interest Ratings at T3 (Current) from All Explanatory Variables

Independent variables (all explanatory)	Dependent variables T3 current interest ratings											
	Academic faculty/research			Academic faculty/teaching			Nonacademic research			Science/nonresearch		
	Unstd. Coeff.	Sig.	Effect size	Unstd. Coeff.	Sig.	Effect size	Unstd. Coeff.	Sig.	Effect size	Unstd. Coeff.	Sig.	Effect size
Variables in the equation												
(Intercept)	2.708	***		2.342	***		2.709	***		2.499	***	
PhD Advisor career advice				0.034								
PhD Advisor career advice-BY-UR status				−0.141	*							
Top 50 undergraduate (UG) institution										−0.111		
Top 50 UG institution-BY-gender										0.118		
Top 50 UG institution-BY-UR										0.266		
Top 50 UG institution-BY-gender-BY-UR status										−1.401	**	
Postdoctoral belonging, laboratory										−0.076	*	
Postdoctoral faculty support, at institution												
Postdoctoral faculty support, outside institution												
Postdoctoral faculty support, outside institution-BY-gender												
Postdoctoral faculty support, outside institution-BY-UR status												
Postdoctoral advisor career advice	0.263	***	S (0.027)									
Years to complete PhD										0.120	**	
Years to complete PhD-BY-gender										−0.163	**	
Confident being independent researcher	0.068	*								−0.049		
Confident being independent researcher-BY-gender										−0.083		
Confident being independent researcher-BY-UR status										−0.209	*	
Confident being independent researcher-BY-gender-BY-UR status										0.411	**	
First-author publication rate										−0.029		
First-author publication rate-BY-UR status										−0.715	*	
(Career aspects) autonomy	0.111	***										
(Career aspects) varied work												
(Career aspects) varied work-BY-gender												
(Career aspects) varied work-BY-UR status												
(Career aspects) varied work-BY-gender-BY-UR status												
(Career aspects) work/life balance	−0.117	***	S (0.01)							0.091	**	
(Features of academia) funding, job market, promotion	0.262	***	S (0.02)				−0.270	***	S (0.038)	−0.202	***	S (0.019)
(Features of Academia) Research, Autonomy	0.495	***	S (0.063)				0.228	***	S (0.021)	−0.127	*	
(Features of academia) research, autonomy-BY-gender										0.177	*	
(Features of academia) teaching, mentoring	−0.144	***		0.560	***	M (0.154)	−0.298	***	S (0.045)			
(Features of academia) work/life balance	0.111	*		0.197	***	S (0.014)						
Gender							0.157	**		−0.259	**	S (0.01)
UR status				0.360	*					0.050		
Gender-BY-UR status										0.201		
Model statistics												
*R*^2^	0.458			0.260			0.090			0.161		
Adjusted *R*2	0.454			0.258			0.088			0.147		
*F*	123.75			103.77			36.62			11.55		
df	9			6			5			21		
df.residual	1173			1473			1474			1204		
*p*	0.0000			0.0000			0.0000			0.0000		

Results of regressions for each of the four career interest T3 ratings (current) on significantly correlated variables (Extended Data Table 3-1) and significant predictor interactions (Extended Data Table 3-2). Unstd. Coeff. = Unstandardized coefficient; sig. = significance. Effect sizes are labeled when they reach at least “small” size. (S) = small effect size, (M) = medium effect size. *p < 0.05; **p < 0.01; ***p < 0.001.

10.1523/ENEURO.0163-21.2021.t3-1 Extended Data Table 3-1 Correlations for T3 Regressions. Correlation of all explanatory variables with current interest ratings. * = p < 0.05, ** = p < 0.01, *** = p < 0.001. Shaded: > 2% variance. Download Table 3-1, DOC file.

10.1523/ENEURO.0163-21.2021.t3-2 Extended Data Table 3-2 Interaction Tests for T3 Regressions. Abbreviated results for investigation of interactions between the explanatory variables and Gender and UR status * = p < 0.05, ** = p < 0.01, *** = p < 0.001. Download Table 3-2, DOC file.

#### Predictors of current interest in research-focused academic positions

**Figure 10.**
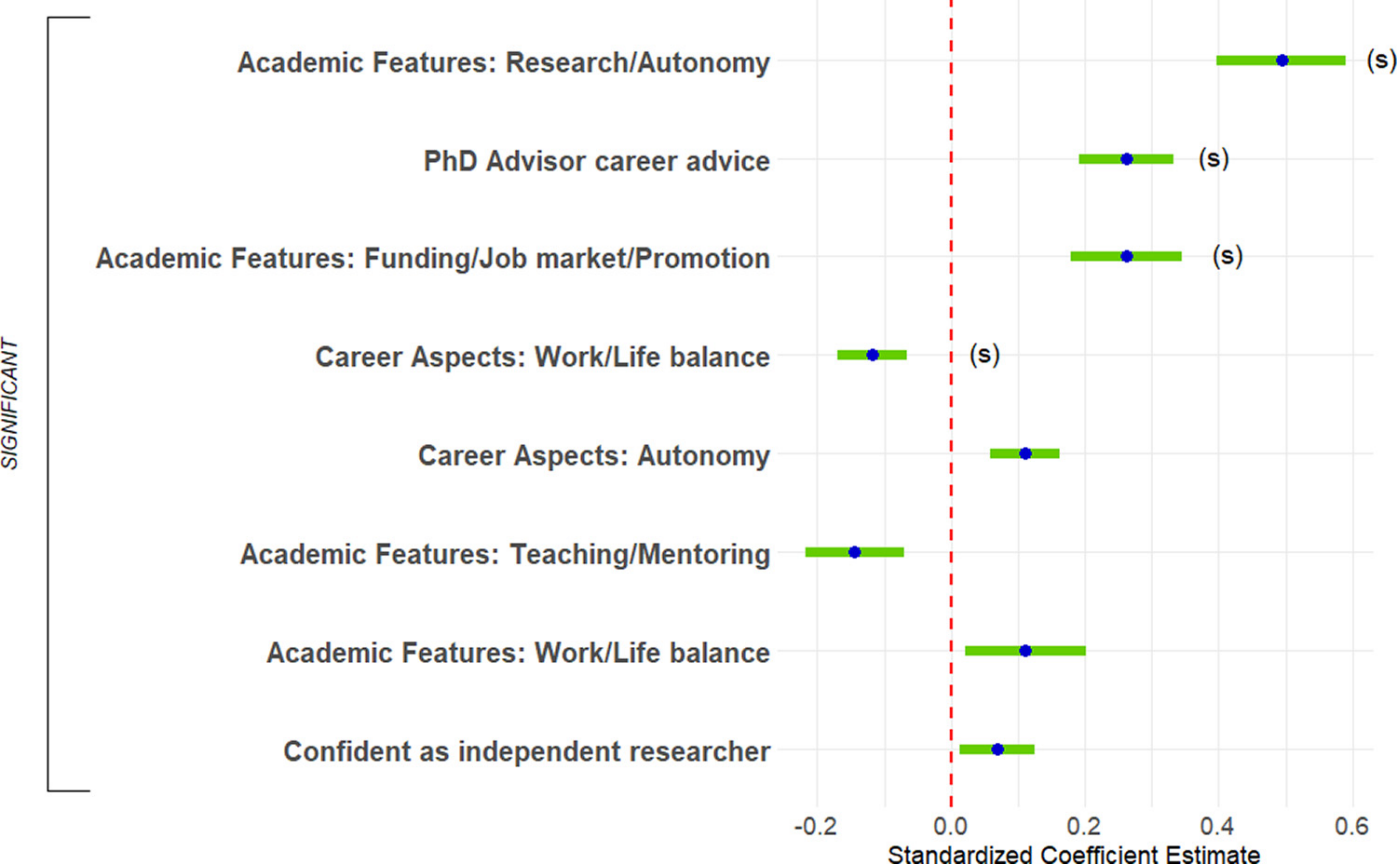
Predicting T3 interest in research-focused academic faculty positions. Standardized regression coefficients and error bars for linear regression predicting current interest (T3) in research-focused academic faculty positions. Dependent variable was current level of interest on a 4-point scale (where 1 represents “no interest” and 4 represents “strong interest”). Independent variables captured experiences during PhD training and postdocs, personal characteristics, objective measures, and interactions with gender and UR status. The entire equation was significant at *p* < 0.001 and captured 45.4% of the variance (adjusted; Table 3). Effect sizes are labeled when they reach at least "small" size. (s) = small effect size.

First, we predicted respondents’ ratings of current interest in research-focused academic faculty positions. The full regression equation was significant, accounting for 45.4% of the variance (Fig. 10, Table 3, adjusted *R*^2^). Four predictors were significant and had sufficient effect size to report. First, more helpful career advice from their PhD advisors was associated with greater interest in academic research positions (2.7% of the variance). Second, respondents for whom work/life balance was less important were more interested in academic research positions (1.0% of the variance). Third, respondents who were more positive about the funding/job market/promotion features of academia were more interested in academic research positions (2.0% of the variance). Finally, respondents who were more positive about the research/autonomy features of academia were more interested in academic research positions (6.3% of the variance). Although other single variables were significant predictors, their effect sizes did not meet the threshold for reporting. In addition, there were no significant interactions for this equation.

#### Predictors of current interest in teaching-focused academic positions

**Figure 11.**
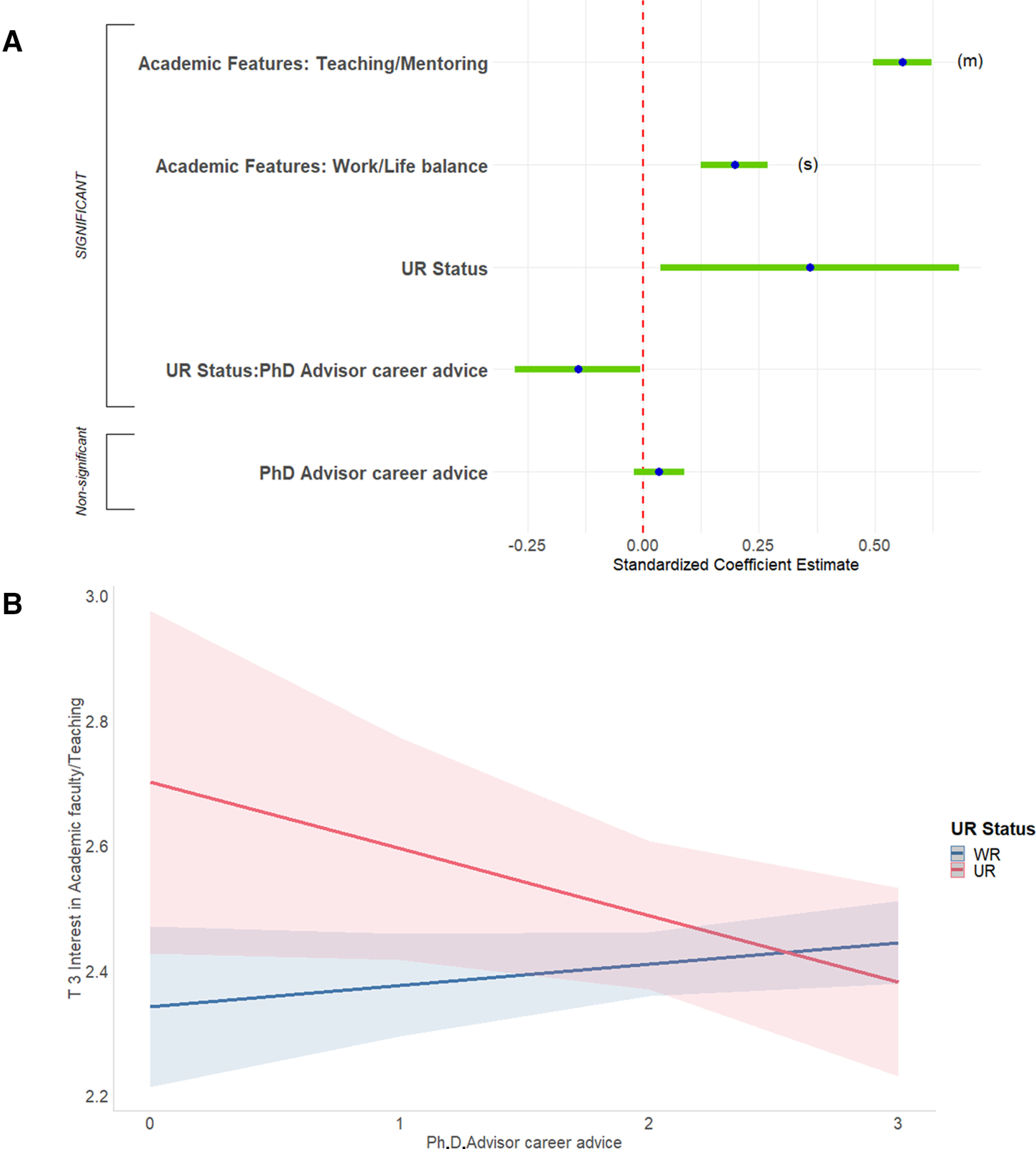
Predicting T3 interest in teaching-focused academic faculty positions. ***A***, Graph shows standardized regression coefficients and error bars for linear regression predicting current interest (T3) in teaching-focused academic faculty positions. Dependent variable was current level of interest on a 4-point scale (where 1 represents “no interest” and 4 represents “strong interest”). Independent variables captured their experiences during PhD training and postdocs, personal characteristics, objective measures, and interactions with gender and UR status. The entire equation was significant at *p* < 0.001 and captured 25.8% of the variance (adjusted; Table 3). Effect sizes are labeled when they reach at least “small” size. (s) = small effect size, (m) = medium effect size. ***B***, Helpful PhD advisor career advice was associated with increased interest in teaching-focused academic faculty positions for WR respondents but was associated with decreased interest in UR respondents. Regression lines predicting current (T3) interest in teaching-focused academic faculty positions from PhD advisor career advice, split by UR status. Dependent was current level of interest on a 4-point scale (where 1 represents “no interest” and 4 represents “strong interest”). Independent variable was helpfulness of career advice from PhD advisor (0–3, 3 being very helpful). Interaction was significant at *p* < 0.05 (Table 3; Extended data Figure 11-1).

Second, we predicted respondents’ ratings of current interest in teaching-focused academic faculty positions. The full regression equation was significant, accounting for 25.8% of the variance (Fig. 11*A*, Table 3, adjusted *R*^2^, Extended data Fig. 11-1). Two predictors were significant and had sufficient effect size to report. First, perhaps unsurprisingly, respondents who were more positive about the teaching/mentoring features of academia were more interested in academic teaching (15.4% of the variance). Second, respondents for whom work/life balance was important were more interested in academic teaching (1.4% of the variance).

10.1523/ENEURO.0163-21.2021.f11-1 Extended Data Figure 11-1 Follow-ups for significant interactions in regressions predicting T3 interest. Follow-up results for significant interactions in the final regressions reported in Table 3. UR = underrepresented, WR = well represented. * = p < 0.05, ** = p < 0.01, *** = p < 0.001. Download Figure 11-1, DOC file.

A single two-way interaction, between UR status and helpfulness of career advice from your PhD advisor, was significant. Helpful career advice from the PhD advisor was weakly associated with increased interest in teaching-focused academic faculty positions for WR respondents but was strongly associated with decreased interest in UR respondents (Fig. 11*B*).

#### Predictors of current interest in nonacademic research positions

**Figure 12.**
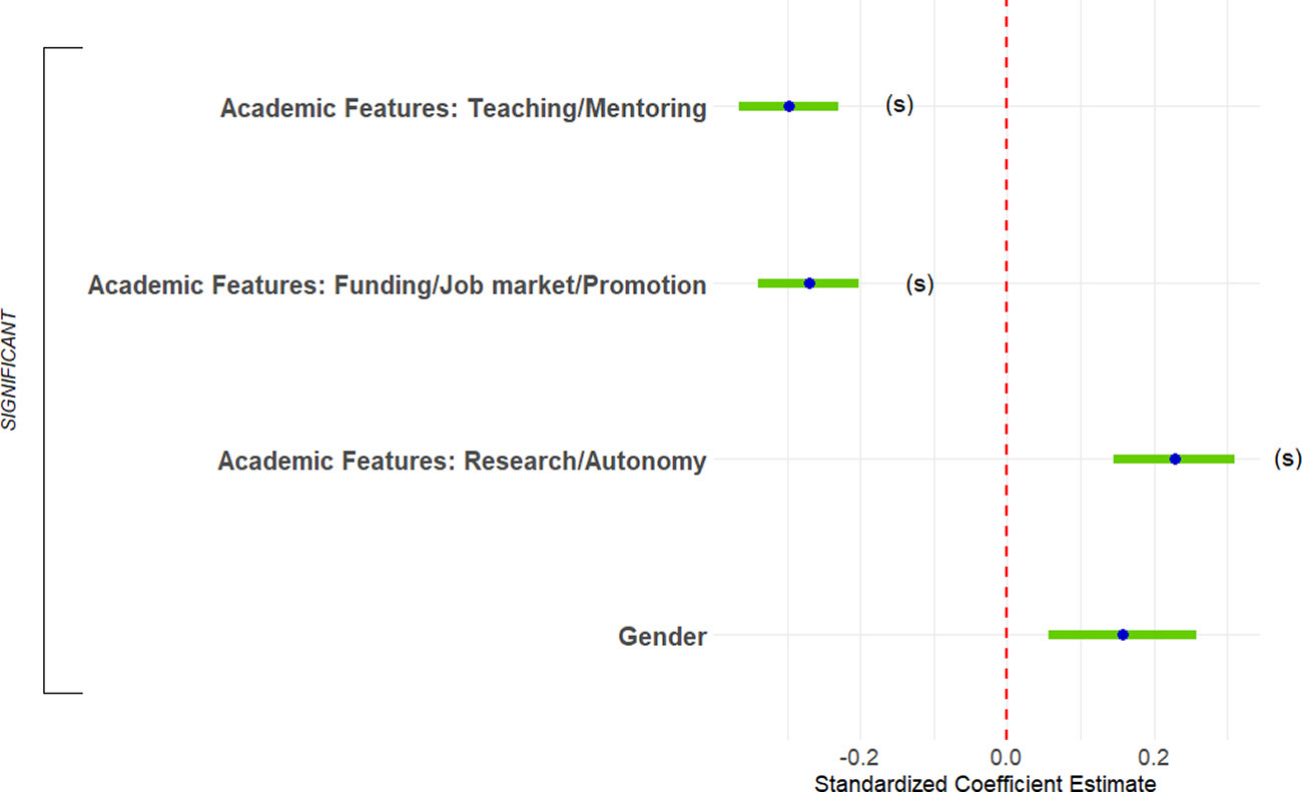
Predicting T3 interest in non-academic research positions. Standardized regression coefficients and error bars for linear regression predicting current interest (T3) in non-academic research positions. Dependent variable was current level of interest on a 4-point scale (where 1 represents “no interest” and 4 represents “strong interest”). Independent variables captured their experiences during PhD training and postdocs, personal characteristics, objective measures, and interactions with gender and UR status. The entire equation was significant at *p* < 0.001 and captured 8.8% of the variance (adjusted; Table 3). Effect sizes are labeled when they reach at least “small” size. (s) = small effect size.

Third, we predicted respondents’ ratings of current interest in nonacademic research positions. The full regression equation was significant but accounted for only 8.8% of the variance (Fig. 12, Table 3, adjusted *R*^2^). Three predictors were significant and had sufficient effect size to report. First, respondents who were more negative about the funding/job market/promotion features of academia were more interested in nonacademic research positions (3.8% of the variance). Second, respondents who were more positive about the research/autonomy features of academia were more interested in nonacademic research positions (2.1% of the variance). Finally, respondents who were more negative about the teaching/mentoring features of academia were more interested in nonacademic research positions (4.5% of the variance). There were no significant interactions for this equation.

#### Predictors of current interest in science-related, nonresearch positions

**Figure 13.**
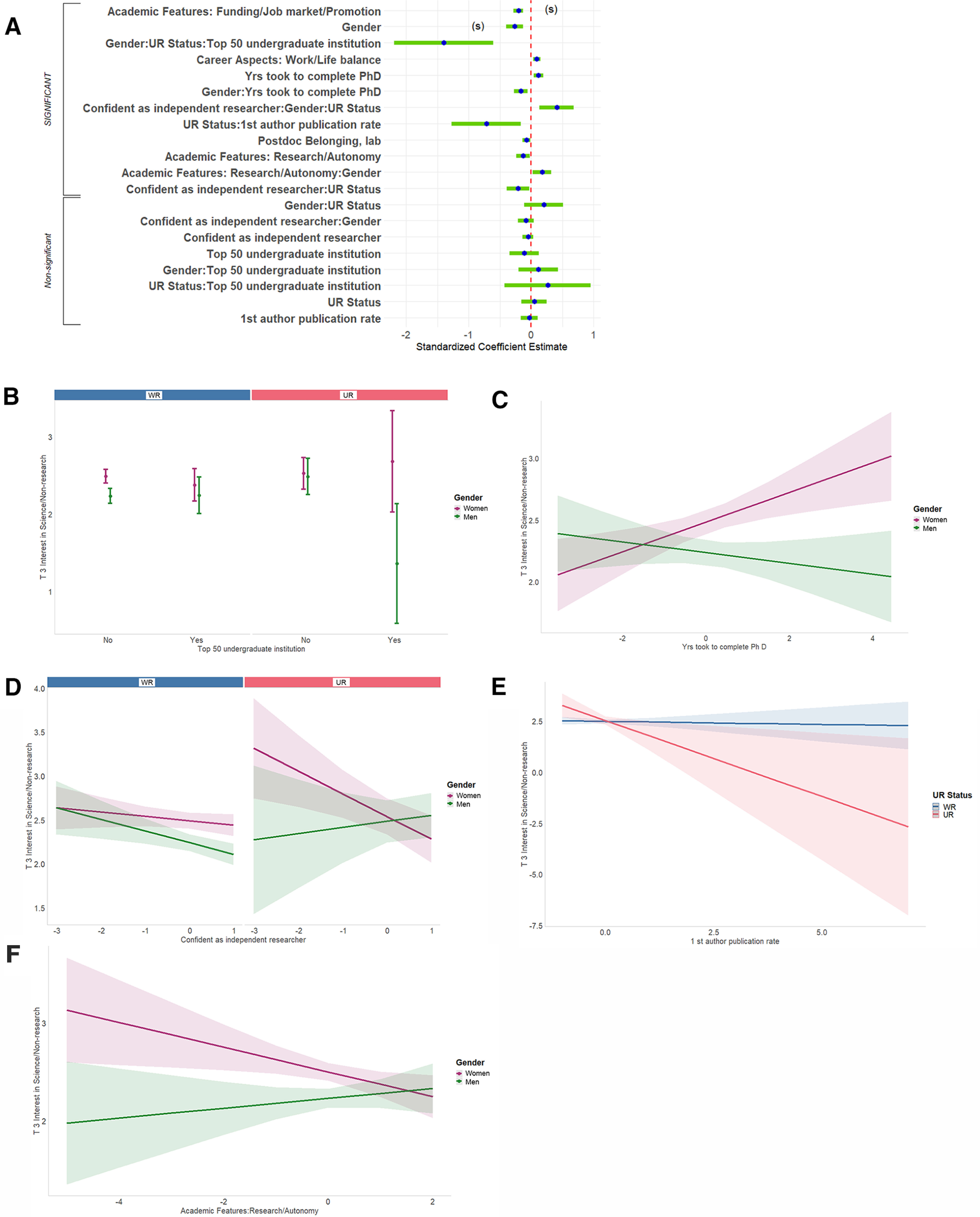
Predicting T3 interest in science-related, non-research positions. ***A***, Standardized regression coefficients and error bars for linear regression predicting current interest (T3) in science-related, non-research positions. Dependent variable was current level of interest on a 4-point scale (where 1 represents “no interest” and 4 represents “strong interest”). Independent variables captured experiences during PhD training and postdoc, personal characteristics, objective measures, and interactions with gender and UR status. The entire equation was significant at *p* < 0.001 and captured 14.7% of the variance (adjusted). Effect sizes are labeled when they reach at least “small” size. (s) = small effect size. ***B***, Top 50 undergraduate institution by gender by UR status interaction predicting T3 interest in science-related, non-research positions. Mean/error lines depicting current (T3) interest in science-related, non-research positions, split by gender and UR status, grouped by whether they had graduated from a top 50 undergrad institution. Dependent variable was current level of interest. Interaction was significant at *p* < 0.01. ***C***, Time to complete PhD was associated with increased interest in science-related, non-research positions for women. Regression lines predicting current (T3) interest in science-related, non-research positions from number of years to complete PhD, split by gender. Dependent variable was current level of interest. Independent variable was number of years to complete PhD, centered for interaction (−4 to +5). Interaction was significant at *p* < 0.01. ***D***, Confidence in being an independent researcher by gender by UR interaction predicting T3 interest in science-related, non-research positions. Regression lines predicting current (T3) interest in science-related, non-research positions from confidence in being an independent researcher, split by gender and UR status. Dependent variable was current level of interest. Independent variable was confidence in being an independent researcher, centered for interaction (-3 to 1). Interaction was significant at *p* < 0.01. ***E***, Lower first author publication rate was associated with increased interest in science-related, non-research positions for UR, but not WR, respondents. Regression lines predicting current (T3) interest in science-related, non-research positions from first author publication rate, split by UR status. Dependent variable was current level of interest. Independent variable was 1st author publication rate, centered for interaction (−1 to +7). Interaction was significant at *p* < 0.05. ***F***, Valuing research/autonomy in academia was associated with a decrease in interest for science-related, non-research positions for women. Regression lines predicting current (T3) interest in science-related, non-research positions from rating of the importance of research/autonomy in academia, split by gender. Dependent variable was current level of interest. Independent variable was factor representing the importance of research/autonomy in academia. Interaction was significant at *p* < 0.05. For ***A–E***, statistics are reported in Table 3; Extended data Figure 11-1.

Finally, we predicted respondents’ ratings of current interest in science-related, nonresearch positions. The full regression equation was significant, accounting for only 14.7% of the variance (Fig. 13*A*, Table 3, adjusted *R*^2^). Two predictors were significant and had sufficient effect size to report. First, respondents who were more negative about the funding/job market/promotion features of academia were more interested in science-related, nonresearch positions (1.9% of the variance). Second, men were less interested in science-related, nonresearch positions (1.0% of the variance).

There were no fewer than six significant interactions for this equation, although one was a two-way interaction within the context of a three-way interaction and will not be reported. The first was a complex three-way interaction among gender, UR status, and whether respondents went to a top 50 undergraduate institution. Two groups of men were less interested in science-related, nonresearch positions than the women in their groups: WR men who were not at a top 50 undergraduate institution, and UR men who were at a top 50 undergraduate institution (Fig. 13*B*)

The second interaction was a two-way interaction between gender and years it took to complete PhD. While for women the longer it took to finish their PhD the more interested in science-related, nonresearch positions they were, for men the longer it took to finish their PhD the less interested in science-related, nonresearch positions they were (Fig. 13*C*). The third interaction was a three-way interaction among gender, UR status, and confidence in their potential to be an independent researcher. While for UR women and WR respondents, lower confidence in their potential to be an independent researcher was related to higher interest in science-related, nonresearch positions, for UR men lower confidence in their potential to be an independent researcher was related to lower interest in science-related, nonresearch positions (Fig. 13*D*).

The fourth interaction was a two-way interaction between UR status and first-author publication rate. While for URs the lower their first-author publication rate, the more interested in science-related, nonresearch positions they were, for WRs there was no association (Fig. 13*E*). Finally, the fifth interaction was a two-way interaction between gender and the features of academia “research/autonomy” factor. While for women, the lower their interest in the research/autonomy features of academia the more interested in science-related, nonresearch positions they were, for men there was no association (Fig. 13*F*).

## Discussion

This work was designed to investigate how career interest evolves over time among recent neuroscience PhD graduates, and whether differences in career interests are related to differences in social identity (gender, race, ethnicity), experiences in graduate school, and personal characteristics. We hypothesized that all three factors would be related to changes in career interest during graduate school and beyond.

Several caveats should be mentioned. First, we analyzed survey results from 1479 US citizen or permanent resident PhD holders who had previously applied for or received NINDS support and obtained their PhD in 2008 or later. Although noncitizens make up a sizeable number of trainees (and the majority of postdoctoral fellows) in the United States (National Science Foundation, 2015); NINDS training and career development awards are generally only available to US citizens during training (with a few exceptions), and thus our analysis was limited to that population. Second, although our response rate of ∼36% of the population (i.e., 45% of opened emails) is in line with other surveys, it is not a random sample. The sample likely has an overrepresentation of people such as those likely to respond to surveys, those with an interest in the topic, and, because of the conventionally public nature of academic contact information, those in academic positions. However, we have a similar sample demographic as other studies (Gibbs et al., 2014). Third, the number of explanatory (independent) variables explored in our analyses was quite large. Steps taken to minimize type I error included false discovery rate control and the use of effect size cutoffs. Fourth, this survey was conducted before the COVID-19 pandemic and does not reflect the likely sizeable influence of this event. Fifth, respondents were asked to retrospectively rate their career interests at the start and end of their PhD training. As such, these ratings represent their current view of their interest in the past. This fits with the goals of the survey, which include understanding respondents’ views of their career trajectories.

A final limitation of our study is that, because of the methods used, our investigation of social identity is narrow in scope and collapses rich, multifaceted characteristics into large enough sample sizes to analyze with regression methods, namely binary gender and binary groups of race/ethnicity (WR and UR respondents) as categorized by the US Government Office of Management and Budget standards. Although we collected information on other marginalized identities, such as disability status, nonbinary gender identity, and sexual orientation, these were not included as separate analysis groups because of small sample sizes. Research on questions about groups that make up a small proportion of a larger community is better served by targeted outreach, snowball sampling, and qualitative or ethnographic research, as opposed to the regressions used in the current work, which require substantial sample sizes. Previous work has shown that these identities face both similar and additional challenges in STEM (science, technology, engineering, and math) advancement, such as lack of accommodations, stigma, and discrimination (da Silva Cardoso et al., 2013; Slaton, 2013; Yoder and Mattheis, 2016; Cech and Waidzunas, 2021).

### Career interest and mentorship

In our sample, we saw the following trends similar to those in other retrospective and cross-sectional studies: interest in academia, both research- and teaching-focused positions, goes down over time, while interest in nonacademic careers goes up over time (Golde and Dore, 2001; Fuhrmann et al., 2011; Goulden et al., 2011; Sauermann and Roach, 2012; Gibbs et al., 2014, 2015; Roach and Sauermann, 2017; but see Wood et al., 2020). However, there are differences between subgroups in our sample, as explored below.

One common factor across all groups is the outsize influence of the academic advisor on career interest. During training, the PhD or postdoctoral advisor can be a source of guidance, support, and networking opportunities (Barnes, 2009). Alternatively, this relationship can also be a source of frustration, disappointment, and discouragement (Thomas et al., 2007; Zhao et al., 2007; Barnes et al., 2010; Barker, 2011; Burt et al., 2020). Previous studies have shown that quality supervision and mentoring are related to persistence in STEM (Berg and Ferber, 1983; de Valero, 2001; Gardner, 2008; Palmer and Young, 2009; Estrada et al., 2018). We found that career advice from PhD advisors perceived as helpful to the trainee is related to increases in interest in academic research and decreases in interest in science nonresearch during graduate school. In addition, helpful career advice for postdoctoral students from their advisors is related to higher current interest in academic research. This emphasizes how effective research mentoring relationships are critical to developing the next generation of researchers. Concrete examples of recommendations, tools, and resources exist to influence the mentor–mentee relationship (Pfund et al., 2015; National Academies of Sciences, Engineering, and Medicine, 2019, 2020a,b, 2021a; Branchaw et al., 2020; Davis et al., 2020; House et al., 2020). Our findings highlight that the key is to implement these strategies with an awareness of equity and inclusion for all trainees (Edwards et al., 2020; Singleton et al., 2021). These strategies are being incorporated into the NINDS strategic plan for 2021–2025 [see: National Institute of Neurological Disorders and Stroke 2021-2026 Strategic Plan (https://www.ninds.nih.gov/About-NINDS/Strategic-Plans-Evaluations/Strategic-Plans/NINDS-Strategic-Plan-and-Priorities)].

### Effect of social identity

#### Gender

The drop in the representation of trainees from marginalized backgrounds over the course of training has commonly been described as the leaky pipeline (McGee et al., 2012; Miller and Wai, 2015), a metaphor that assumes that at PhD program entry all trainees aspire to a faculty research position. In contrast, we found that not only do some individuals enter graduate school with low interest in faculty research positions (and this is the biggest single predictor of interest at the end of PhD programs), but also that interest may vary by social identity group. In particular, women reported being less interested in academic research careers at the start of their PhD—and their interest drops more over time—and are correspondingly more interested in science nonresearch at the start of their PhD and gain more interest over time compared with their men counterparts.

What may explain these differences? In the realm of individual preferences, women in our study report a higher importance of work/life balance as an aspect of their careers. This is in line with a large body of research that has found that work/family balance challenges influence women graduate students and postdoctoral students away from faculty careers (Martinez et al., 2007; Sassler et al., 2017; Lambert et al., 2020). Beddoes and Pawley (2014) found that faculty identified similar family-related challenges as contributing to low numbers of women STEM faculty. However, they also stressed that the discourse of “personal choice” within an inequitable situation can obscure the systemic pressures that different groups face. For example, in man–woman partnerships, a disproportionate amount of family and household responsibilities falls to women who work full-time (Schiebinger and Gilmartin, 2010; also see https://www.americanprogress.org/issues/women/reports/2018/05/18/450972/unequal-division-labor/), and, in particular, the difficulties of balancing caregiving responsibilities with full-time work in STEM may explain why women are more likely than men to leave academia or STEM employment entirely after having children (Wolfinger et al., 2008; Goulden et al., 2011; Martinez et al., 2017; Cech and Blair-Loy, 2019) and may contribute to lower publication rates among women (Morgan et al., 2021). This has never been in sharper relief than during the current pandemic, which has disproportionately affected women, especially women with young children (Gewin, 2020; Kramer, 2020; Myers et al., 2020; National Academies of Sciences, Engineering, and Medicine, 2021b). These issues are further compounded for women of color in academia (Kachchaf et al., 2015; Guy and Boards, 2019). As Beddoes and Pawley (2014) point out, more important than describing the choices that are made is understanding why other careers are more appealing than academic research for certain groups, instead of “writing it off as a matter of individual priorities.” In our study, among all respondents, rating work/life balance as important led to lower interest in academic research and higher interest in academic teaching. Thus, it is not academia itself that is considered incompatible with work/life balance, but rather something unique to academic research-focused positions rather than teaching-focused positions.

Institutions are beginning to recognize that challenges surrounding work/life balance exist and differentially affect mothers in science, including the exorbitant cost of childcare and navigating frequent travel to conferences (Calisi, 2018; although, for a discussion of the lack of support for childless women in academia, see Cummins, 2005). To that end, some universities offer subsides and other forms of support to working parents (see programs at University of Iowa, Brown University, Stanford University, University of Michigan, University of Massachusetts Amherst and more). NIH has several programs and policies to support working parents, as follows: (1) most recently, trainees and fellows supported by National Research Service Awards may now request support for childcare costs [see Announcement of Childcare Costs for Ruth L. Kirschstein National Research Service Award (NRSA) Supported Individual Fellows (https://nexus.od.nih.gov/all/2021/03/02/announcement-of-childcare-costs-for-ruth-l-kirschstein-national-research-service-award-nrsa-supported-individual-fellows/)]; (2) Early Stage Investigator status and K99/R00 eligibility is automatically extended by a year for individuals who have had a child after completing their terminal degree, and extensions are available for other life events [see Update on NIH Extension Policy for Early Stage Investigator Status (ESI), Notice Number NOT-OD-18-235 (https://grants.nih.gov/grants/guide/notice-files/NOT-OD-18-235.html) and NIH Extension Policy for Eligibility Window for Pathway to Independence Awards (K99/R00), Notice Number NOT-OD-20-011 (https://grants.nih.gov/grants/guide/notice-files/NOT-OD-20-011.html)]; and (3) NIH has made supplements available to primary investigators of R and K awards who have experienced critical life events (such as childbirth, adoption, or elder care) that have the potential on impact research progress [see Administrative Supplements to Promote Research Continuity and Retention of NIH Mentored Career Development (K) Award Recipients and Scholars, NOT-OD-20-054 (https://grants.nih.gov/grants/guide/notice-files/not-od-20-054.html) and Notice of Special Interest (NOSI): Administrative Supplement for Continuity of Biomedical and Behavioral Research Among First-Time Recipients of NIH Research Project Grant Awards, Notice Number, NOT-OD-20-055 (https://grants.nih.gov/grants/guide/notice-files/NOT-OD-20-055.html)]. More acutely, the COVID-19 pandemic has put into sharp relief both the tensions and some possible solutions for managing work–life integration (Malisch et al., 2020). Additional policies and programs in this area may have a positive impact, not only on women’s persistence in academic research, but on the culture of work–life integration in science, allowing scientists to navigate many of life’s challenges, such as medical events or other emergency circumstances.

In our survey, women also report that the structural aspects of academia (grant funding, job market, promotion) reduce their interest in academia more than they do for men. Among the entire sample, more negative feelings about the structural aspects of academia (funding/job market/promotion) were related to lower interest in academic and nonacademic research and higher interest in science-related nonresearch careers. While increasing support for parents provides a clear area of intervention, structural issues around scarce funding and attendant issues such as the job market and promotion are harder to ameliorate. However, one potential lever within discussions of structural aspects of academia is service, which can be to the department, university, or professional society and include tasks such as governance, faculty recruitment, and student admissions. While these activities are factored into the consideration of promotion and tenure, service is typically valued less than research (and/or teaching, depending on the institution type). Women and/or UR faculty take on disproportionate amounts of service when considering both the number of activities and the time spent (Bird et al., 2004; Whittaker et al., 2015; Guarino and Borden, 2017). This represents time spent away from activities that support more valued work such as research and securing grant funding. Institutions may also consider the distribution of service tasks and the value assigned to this necessary work in promotion and tenure policies. NINDS has directed particular attention toward supporting junior faculty in the hopes of easing structural concerns, through the Early Stage Investigator policy, postdoctoral–faculty transition awards (K99/R00), the K01 diversity faculty award (which, like all Ks, provides 75% protected time), research education awards (R25s) for career development of junior faculty, and participation in the NIH Faculty Institutional Recruitment for Sustainable Transformation (FIRST) Program to support cohort hiring of scientists committed to diversity and inclusive excellence, among other efforts.

In addition to differences in their ratings of interest in the different career types, women reported issues that may be roadblocks to academic research careers, such as poorer relationships with their PhD advisors, lower first-author publication rates, and less confidence in their ability to be independent researchers compared with men. For UR women, in particular, having lower confidence in their ability to be an independent researcher is related to higher interest in science-related nonresearch. The impact of bias, exclusion, and structural racism likely contribute to these findings and can become the drivers for what is commonly known as “imposter syndrome,” a controversial term that centers the problem in the individual, rather than the system (Tulshyan and Burey, 2021). These results mirror the findings of other studies (Martinez et al., 2007; Cheryan et al., 2017) and may stem either from women underestimating their ability or men being overconfident in their ability to be independent researchers (Bench et al., 2015). Confidence in one’s ability to perform a task, self-efficacy, has been shown to be an important piece of persistence in STEM careers (Berg and Ferber, 1983; Byars-Winston et al., 2015).

#### UR status

Members of marginalized groups also report factors that are roadblocks for academic research careers. For example, UR respondents reported lower first-author publication rates. This finding is in line with other work showing lower numbers of publications for women and/or UR scientists (Pezzoni et al., 2016; Mendoza-Denton et al., 2017; Lambert et al., 2020). For UR respondents, having a lower publication rate is related to a higher interest in nonresearch science careers. This analysis cannot determine the directionality of this association, but the relationship is likely complicated—that is, experiences and perceptions in graduate school influence publication rate and career interests (Fisher et al., 2019), while career interests in turn may influence publication rate. There is also evidence of systemic bias within publishing, as women are consistently underrepresented in scientific journals as authors, in citation counts, and in reference lists (Larivière et al., 2013; Caplar et al., 2017; Shen et al., 2018; Dworkin et al., 2020), and white authors are overrepresented among citation lists (Bertolero et al., 2020). Journals are increasingly turning toward new models to address these findings, such as double-blind review (Bernard, 2018) and inclusion and diversity statements for journal submissions (Sweet, 2021).

Consistent with similar studies, our study shows UR respondents feel less like they were a part of the social and intellectual community during both their PhD and postdoctoral training (Fisher et al., 2019; Stachl and Baranger, 2020). This finding is not particularly surprising, especially in light of the poignant personal stories recently shared from #BlackintheIvory hashtag on Twitter and other venues that illuminate this lack of inclusion in the research enterprise (Armstrong et al., 2020; Dzirasa, 2020; Karanja et al., 2020; Odekunle, 2020). Sense of belonging has been shown to mediate desire to pursue further studies in a discipline (Gardner, 2008; Good et al., 2012), and a feeling of isolation is related to higher attrition rates from PhD programs (Lovitts, 2001; Herzig, 2004b). Conversely, institutional attention to both STEM culture and institutional climate has the potential to enhance persistence in science (see https://www.higheredtoday.org/2018/04/23/addressing-stem-culture-climate-increase-diversity-stem-disciplines/). A fundamental part of the adoption of an identity of a scientist is participation in and integration with a community of practice during academic training (Herzig, 2004a). Barriers to full participation in the social and intellectual community of the PhD and postdoctoral training can have a profound effect on the development of scientific identity. These barriers may be explicit, in the form of hostile or racist work environments or lack of access to professional development resources or mentorship, or may be implicit, in the form of differing values (Gibbs and Griffin, 2013; Aelenei et al., 2020) or alienating cultural assumptions such as a culture perceived as masculine, focused on self-enhancement, or individualistic (Herzig, 2004a; Cheryan et al., 2017; Aelenei et al., 2020). Supporting the latter possibility, members of both underrepresented groups and women in our study report a lower importance of autonomy in their careers, and, among the entire sample, negative feelings about the “autonomy/research” aspects of academia were related to lower interest in research careers. As the importance of team science increases in biomedicine, institutions may rethink models or messaging around the idea of the “lone genius” in favor of collaborative structure and recognition (Bennett et al., 2018). Along those lines, NIH has recently made more explicit calls for team research in investigative neuroscience at different stages and on various scales (David et al., 2020).

The lack of community felt at their home institution may drive UR trainees to find support elsewhere, as demonstrated by reports of beneficial relationships with faculty outside their PhD institutions (Burt et al., 2019; also see http://web.archive.org/web/20160620200254/www.diversityweb.org/Digest/F00/graduate.html). Our study shows that faculty from other institutions had a strong influence on UR respondents. These differences were even more pronounced in UR women during their PhD training compared with UR men, suggesting an intersectional or compounding effect of multiple marginalized social identities (Ong et al., 2011; Guy and Boards, 2019). This result also may speak to the success of networking and professional development programs for underrepresented scientists. NINDS supports programs that strive to equip underrepresented scientists with the tools needed to successfully navigate the challenges of academic life and leverage national peer support systems and mentoring networks outside of the primary institution (Jones-London, 2020). These NINDS-funded programs include the Society for Neuroscience Neuroscience Scholars Program (National Research Council, 2013); the BRAINS program (Margherio et al., 2016; Yen et al., 2017); the TRANSCENDS Program (Tagge et al., 2021); and the NIH Blueprint-funded ENDURE and D-SPAN Programs (Jones-London, 2020).

Together, our findings speak to systemic issues within academia when it comes to supporting scientists from marginalized groups (Griffin, 2019; also see https://www.higheredtoday.org/2018/04/23/addressing-stem-culture-climate-increase-diversity-stem-disciplines/). This includes lack of role models, poorer mentorship, unequal distribution of labor, isolation, gendered and racialized stereotypes contributing to an unwelcoming climate, and disconnection from the perceived values of academic culture. The NIH FIRST program, the NSF ADVANCE program, the AAAS (American Association for the Advancement of Science) SEA (STEM Equity Achievement) Change program, and the Howard Hughes Medical Institute Inclusive Excellence programs, among others, are attempting to increase inclusion and equity by changing the institutional culture, instead of focusing on “fixing” the individual. Strategies include climate assessment, pay equity analysis, changes in faculty recruitment and student admissions strategies, re-evaluation of tenure and promotion policies, and rethinking institutional culture (Whittaker et al., 2015; Griffin, 2019).

### Conclusion

We saw repeated evidence that individual preferences about careers in general, and academic careers specifically, predict current career interest. These preferences were moderated by social identity and experiences in graduate school and postdoctoral training. Our findings highlight the outsized influence of the advisor in shaping a trainee’s career path, and the ways in which academic culture is perceived as unwelcoming or incongruent with the values or priorities of certain groups. For women, issues of work/life balance and structural issues of academia, and, for UR women in particular, lower confidence in their ability to be an independent researcher, affected their interest in academia. Both women and underrepresented men in our study report a lower importance of autonomy in their careers. UR respondents report feeling less like they were a part of the social and intellectual community but have formed beneficial relationships with faculty outside their PhD institutions that are—for UR women—associated with increased interest in academia. Although the effect sizes are mostly modest for these findings, the results do not reflect shared variance with other variables in the analysis, and likely underestimate their true influence. Our findings suggest several areas for positive growth, ways to change how we think about the impact of mentorship, and policy and programmatic interventions that extend beyond trying to change or “fix” the individual and instead recognize the systemic structures that influence career choices. Importantly, we recognize that not all holders of a PhD will or should express interest in academic research, but that career path should be equally available and welcoming to all so that our nation can benefit from the full potential of its diverse workforce.

## Synthesis

Reviewing Editor: Alexxai Kravitz, Washington University in St. Louis Decisions are customarily a result of the Reviewing Editor and the peer reviewers coming together and discussing their recommendations until a consensus is reached. When revisions are invited, a fact-based synthesis statement explaining their decision and outlining what is needed to prepare a revision will be listed below. The following reviewer(s) agreed to reveal their identity: Julio Ramirez, Kaela Singleton.

Your manuscript was seen by two reviewers who were both very positive on it and thought it should be published quickly. However, they make a number of points to improve it, with some suggesting additional clarity in the figures, and others making suggestion for language, or additional discussion of points that were made. I have pasted their full comments below and we discussed and came to the agreement that 1) their points should be addressed in full, but 2) no large changes have been requested and all points can be addressed by changing or enhancing the writing.

There is one exception to this, which is to possibly analyze the data based on sexual orientation as it appears that data may have been collected but not discussed (see reviewer 2 below). I look forward to seeing your revised manuscript.

Full comments:

**********************

Reviewer 1

**********************

The article entitled “Factors that Influence Career Choice Among Different Populations of Neuroscience Trainees” is an excellent analysis of the personal and social factors that operate to affect career choices among well-represented (WR) and underrepresented (UR) neuroscience trainees who have been supported by the NINDS. The introduction presents a thorough review and logical argument for the importance of undertaking this project. The Methods section clearly describes how the project was conducted from data sets collected at the NINDS, which is a unique position to undertake such a large-scale and comprehensive assessment of young neuroscientists comprising its awardee pool. The refinement procedures described instill confidence in the adequacy and representativeness of the sample finally arrived at for the undertaking of this study. The size of each it subset of participants is reasonable given the larger pool from which the final sample was drawn and it is noteworthy that 16% of the sample was from the UR trainee group and 54% from female trainees. Because the paucity of awardees reporting a disability, the author(s) rationale for not assessing this group is reasonable. Of course, the fact that so few respondents from the disabled community were available is in itself a notable issue. The statistical methodology deployed to assess their data was thorough and sound. Overall, the findings are novel and compelling. Understandably, given the complexity of this multifactor assessment, the outcomes are multifaceted and sometimes surprising, if not counterintuitive at times. For example, this reviewer was struck by the observation that whereas UR females and WR participants who expressed lower confidence in their potential to be an independent research also reported a higher interest in in science-related, non-research positions, UR males reported both a lower level of confidence and lower interest in science-related, non-research positions. The Discussion section does an excellent job of integrating their observations into the broader fabric of this research area, which is frankly only now becoming more actively investigated. Importantly, the observations made here provide excellent points for NINDS and higher education institutions to consider for intervention in order to promote equity and accessibility across the breadth of biomedical science training programs.

Overall, this manuscript is suitable for publication in eNeuro after minor revision and the author(s) are to be commended for undertaking this assessment.

Points to consider:

1)The principal shortcoming of this study was the retrospective nature of the data collection. The participants were asked at one point in time to reflect on the level of interest from points in the past. A stronger approach would have of course been a longitudinal study that followed the participants throughout the period of interest. That said, the approach adopted herein is an accepted method in survey-type research, to the best of this reviewer’s knowledge.

2)Although the assessment of the undergraduate origins of the participants is most certainly an important issue to explore. The top 50 undergraduate institutions explored in this study were all research universities. Two other undergraduate institution types would have been especially interesting to consider: minority serving institutions (i.e., HBCUs and HSIs) as well liberal arts colleges. These institutions are known to produce a disproportionate number of PhDs in the sciences and engineering, so this information could have been useful in the context of this study.

Minor recommendations:

1)The color schemes in figures such as 13B (blue header with black letters and red header with black letters) are very difficult to read. They should be made more legible.

2)Sub-headings would be helpful as the readers makes their way through a number of sections, for example those sections focused on observations for women participants or those from UR participants.

**********************

Reviewer 2

**********************

Overall, this manuscript is a great read! I commend the authors for presenting clear and concise data as well as explaining the current state of the Neuroscience community. Further, they do an excellent job explaining the programs currently underway to support minoritized/marginalized trainees. The following are facets of the manuscript that need to be updated:

Significance Statement/Broad Conclusions: The current manuscript expands and uses data to characterize the experience of female vs male early career scientists. Importantly, more concrete descriptions of how and what “areas for positive growth, ways to change how [we] think about the impact of mentorship” need to be included for the significance statement within the text to be true. Explicit statements within the discussion showing how and what “areas for positive growth, ways to change how we think about the impact of mentorship” need to be included for this conclusion to be true. Several articles from Neuroscience Nature and Cell Press provide potential solutions that can be cited and included within the manuscript.

Gender vs Sex: Throughout the manuscript and figures (and within the survey itself) authors use gender and biological sex interchangeably and/or confuse the terms. Gender is a social construct and often referred to as a binary - man or woman, while Sex (or biological sex) refers to the genitals you’re born with and the chromosomes you have (male vs female). For example, in the section “Differences by Gender and UR in Explanatory Variables” authors use the term female to represent women. If the goal of the manuscript is to evaluate the facets of academia that encourage early career scientists to pursue careers in academia - fixing and acknowledging this error within the text and figures is critical.

Figures: For graphs similar to Figure 7B the text within the blue and pink bars, above regression line graphs are hard to read and should be fixed.

Race/Ethnicity: This comment is for the sake of inclusion and does not warrant a change by the authors. Within the text of the manuscript, authors state that they aim to establish how social identity including gender, race, and ethnicity play a role in career decisions. While authors used NOT-OD-15-089 to classify race/ethnicity, expanding on the limitations of this (within the discussion) could be useful, but is not necessary.

Within limitation section of the discussion authors should indicate why they did not include genders such as trans or non-binary within the survey and address how that impacted their findings. Exclusion of these identities (though considered ‘okay’ due to NIH guidelines) erases a demographic of early career scientists within the field. It also speaks to the issue within the manuscript of Biological Sex vs Gender. Additionally, authors should address if the study asked about sexual orientation. Extended data reveal authors did ask this question, but there is no mention of results. A point within the discussion citing articles that discuss the non-binary and LGBT+ community is necessary to be both inclusive and acknowledge limitations of the study and current programs.

Lines 420-454: While there is a solid explanation of the issue regarding women and work-life balance, as well as support available, authors fail to explicitly connect this issue to increased ‘uncounted’ work women in STEM do. This can include service, mentorship and teaching. It is critical to point out that while STEM/academia are working to become more equitable for women (something the authors do very well!), it is also important to acknowledge the extra burdens put on women, especially women of color, within institutions.

Line 473-473: I really appreciate the phrasing of imposter syndrome phenomena. This would be a good transition into discussing the systemic principles of academia that fail marginalized groups, and the way funding institutions are attempting to improve that (again, authors describe the resources available very well).

## Author Response

Dear Dr. Kravitz,

Thank you for the opportunity to submit a revised draft of the manuscript titled, “Factors that Influence

Career Choice Among Different Populations of Neuroscience Trainees.”

We thank the reviewers for their thoughtful and insightful comments. We have incorporated changes to

reflect the suggestions provided by the reviewers and have highlighted the changes within the

manuscript.

Below is a point-by-point response to the reviewers’ comments:

Reviewer 1

The principal shortcoming of this study was the retrospective nature of the data collection. The

participants were asked at one point in time to reflect on the level of interest from points in the past. A

stronger approach would have of course been a longitudinal study that followed the participants

throughout the period of interest. That said, the approach adopted herein is an accepted method in

survey-type research, to the best of this reviewer’s knowledge.

Response: We agree that this is a significant limitation of our dataset. We still believe there is

meaningful information to be gathered from the retrospective survey, as it represents the participants’

current understanding of their career path to date, but longitudinal information would be more

informative about their contemporary understanding of decisions as they were made.

Although the assessment of the undergraduate origins of the participants is most certainly an important

issue to explore. The top 50 undergraduate institutions explored in this study were all research

universities. Two other undergraduate institution types would have been especially interesting to

consider: minority serving institutions (i.e., HBCUs and HSIs) as well liberal arts colleges. These

institutions are known to produce a disproportionate number of PhDs in the sciences and engineering,

so this information could have been useful in the context of this study.

Response: Thank you for pointing this out. Among our respondents, we had 181 respondents (12%) who

attended MSIs for their undergraduate education (the full classification is available in Table 1). Although

there is no formal list of liberal arts colleges, we have included the Carnegie Classification in Table 1. 216

respondents (15%) attended undergraduate institutions classified as “Baccalaureate Colleges: Arts &

Sciences Focus.” This information has been added to the manuscript in Table 1.

The color schemes in figures such as 13B (blue header with black letters and red header with black

letters) are very difficult to read. They should be made more legible.

Response: We apologize for the illegibility of the header color scheme. The headers for figures 6B, 7B,

13B, and 13D have been revised to improve legibility.

Sub-headings would be helpful as the readers makes their way through a number of sections, for

example those sections focused on observations for women participants or those from UR participants. 2

Response: Apologies for the dense text. Additional subheadings have been added to improve readability,

particularly in the discussion section.

Reviewer 2

Significance Statement/Broad Conclusions: The current manuscript expands and uses data to

characterize the experience of female vs male early career scientists. Importantly, more concrete

descriptions of how and what “areas for positive growth, ways to change how [we] think about the

impact of mentorship” need to be included for the significance statement within the text to be true.

Explicit statements within the discussion showing how and what “areas for positive growth, ways to

change how we think about the impact of mentorship” need to be included for this conclusion to be true.

Several articles from Neuroscience Nature and Cell Press provide potential solutions that can be cited

and included within the manuscript.

Response: Thank you for these comments. We have edited the mentorship section of the discussion to

be explicit about mentorship solutions and future directions.

Gender vs Sex: Throughout the manuscript and figures (and within the survey itself) authors use gender

and biological sex interchangeably and/or confuse the terms. Gender is a social construct and often

referred to as a binary - man or woman, while Sex (or biological sex) refers to the genitals you’re born

with and the chromosomes you have (male vs female). For example, in the section “Differences by

Gender and UR in Explanatory Variables” authors use the term female to represent women. If the goal

of the manuscript is to evaluate the facets of academia that encourage early career scientists to pursue

careers in academia - fixing and acknowledging this error within the text and figures is critical.

Response: Thank you for pointing this out. Our survey question asked about gender, and we have

changed all references to “female” and “male” to “woman” and “man,” where appropriate, to reflect

this. The survey question itself used “male” and female” as response options for gender. This is a

reflection of older conventions used in previous surveys that use “male” and “female” to refer to gender

identity, upon which we based our questions (e.g., Yoder and Mattheis (2015) “Queer in STEM:

Workplace Experiences Reported in a National Survey of LGBTQA Individuals in Science, Technology,

Engineering, and Mathematics Careers”). We recognize that “male” and “female” are more

appropriately used for sex, and have updated the manuscript accordingly.

Figures: For graphs similar to Figure 7B the text within the blue and pink bars, above regression line

graphs are hard to read and should be fixed.

Response: We apologize for the illegibility of the header color scheme. The headers for figures 6B, 7B,

13B, and 13D have been revised to improve legibility.

Race/Ethnicity: This comment is for the sake of inclusion and does not warrant a change by the authors.

Within the text of the manuscript, authors state that they aim to establish how social identity including

gender, race, and ethnicity play a role in career decisions. While authors used NOT-OD-15-089 to classify

race/ethnicity, expanding on the limitations of this (within the discussion) could be useful, but is not

necessary.

Response: Thank you for this comment. We have added a point to the limitations section acknowledging

the narrowness of our definition of social identity.3

Within limitation section of the discussion authors should indicate why they did not include genders

such as trans or non-binary within the survey and address how that impacted their findings. Exclusion of

these identities (though considered ‘okay’ due to NIH guidelines) erases a demographic of early career

scientists within the field. It also speaks to the issue within the manuscript of Biological Sex vs Gender.

Additionally, authors should address if the study asked about sexual orientation. Extended data reveal

authors did ask this question, but there is no mention of results. A point within the discussion citing

articles that discuss the non-binary and LGBT+ community is necessary to be both inclusive and

acknowledge limitations of the study and current programs.

Response: Thank you for your comment. To clarify, as indicated in “Definitions and Sample Refinement,"

transgender participants were not excluded from participating in the survey if they identified as either a

man or woman; if so, they were included within those respondent groups. Participants could indicate

their preferred gender through the write-in option. The two participants who identified as neither man

nor woman were excluded because the sample size was too small to analyze separately. This is in line

with previous studies, where respondents answering “other” or “transgender” make up a very small

percentage of participants when sampling the broad STEM field (e.g., 13 out of 8,099 individuals or 0.16%

in Layton et al (2017) “Diversity Exiting the Academy: Influential Factors for the Career Choice of Well-Represented and Underrepresented Minority Scientists”). Based on previous research, we did not

anticipate having a large enough sample to draw conclusions on using the regression methods in the

paper, and thus did not ask participants to explicitly identify as, e.g., “transgender man” or “transgender

woman,” unless they preferred to do so through the write-in option. Research on questions specifically

about groups that make up a small proportion of the larger community is better served by targeted

outreach, snowball sampling, and qualitative or ethnographic research, which is outside the scope of

this paper. This point has been clarified in the manuscript.

Data were collected on sexual orientation. 1,324 participants identified as “straight or heterosexual,"

while 87 participants identified as “lesbian, gay, homosexual, bisexual, or other sexual minority.” While

the sample size is too small to include as a separate group within our analysis of race and gender, these

results have been added to the demographic information in Table 1 and consideration of the non-binary

and LGBT+ community has been added to the discussion.

Lines 420-454: While there is a solid explanation of the issue regarding women and work-life balance, as

well as support available, authors fail to explicitly connect this issue to increased ‘uncounted’ work

women in STEM do. This can include service, mentorship and teaching. It is critical to point out that

while STEM/academia are working to become more equitable for women (something the authors do

very well!), it is also important to acknowledge the extra burdens put on women, especially women of

color, within institutions.

Response: Thank you for pointing out this oversight. This important consideration has been added to the

discussion section on structural aspects of academia.

Line 473-473: I really appreciate the phrasing of imposter syndrome phenomena. This would be a good 4

transition into discussing the systemic principles of academia that fail marginalized groups, and the way

funding institutions are attempting to improve that (again, authors describe the resources available very

well).

Response: We have added and expanded on the topic of structural change in the last paragraph of the

discussion.

We look forward to hearing from you regarding our submission and to respond to any further questions

and comments you may have.

Sincerely,

Michelle Jones-London, Lauren Ullrich, and John Ogawa
