# Supplementary material for: Factors That Influence Career Choice among Different Populations of Neuroscience Trainees
Source: eNeuro. 2021 Jun 18;8(3):ENEURO.0163-21.2021. doi: 10.1523/ENEURO.0163-21.2021 (PMC8223496; doi:10.1523/ENEURO.0163-21.2021)
Supplement: Extended Data Table 1-1 — Descriptive information for all variables. Descriptive statistics for all dependent and independent variables in the study, by category of variable. Min = minimum value, Max = maximum value, N = number in group, SD = standard deviation. Download Table 1-1, DOC file. [file enu-eN-SIM-0163-21-s06.doc]

|  |  |  | |  | |  |  | | |  |
| --- | --- | --- | --- | --- | --- | --- | --- | --- | --- | --- |
| **Dependent Variable Career Interest Ratings** | **N** | **Min** | | **Max** | | **Median** | **Mean** | | | **SD** |
| ***(At start of PhD)*** | | | | | | | | | | |
| Academic Faculty/Research | 1479 | 1 | | 4 | | 4 | 3.58 | | | 0.7000 |
| Academic Faculty/Teaching | 1479 | 1 | | 4 | | 3 | 2.75 | | | 0.9200 |
| Non-academic Research | 1479 | 1 | | 4 | | 2 | 2.50 | | | 0.9300 |
| Science/Non-research | 1479 | 1 | | 4 | | 2 | 1.91 | | | 0.8300 |
| ***(At end of PhD)*** | | | | | | | | | | |
| Academic Faculty/Research | 1479 | 1 | | 4 | | 4 | 3.26 | | | 0.9800 |
| Academic Faculty/Teaching | 1479 | 1 | | 4 | | 3 | 2.56 | | | 0.9800 |
| Non-academic Research | 1479 | 1 | | 4 | | 3 | 2.69 | | | 0.9600 |
| Science/Non-research | 1479 | 1 | | 4 | | 2 | 2.20 | | | 1.0000 |
| ***(Current)*** | | | | | | | | | | |
| Academic Faculty/Research | 1479 | 1 | | 4 | | 3 | 2.99 | | | 1.1700 |
| Academic Faculty/Teaching | 1479 | 1 | | 4 | | 2 | 2.43 | | | 1.0500 |
| Non-academic Research | 1479 | 1 | | 4 | | 3 | 2.78 | | | 1.0200 |
| Science/Non-research | 1479 | 1 | | 4 | | 2 | 2.41 | | | 1.0700 |
| **Graduate School Era Explanatory Variables** |  |  | |  | |  | | |  |  |
| **Independent Variable Predictors of T1 (Start PhD) and T2 (End PhD) interest** | **N** | **Min** | | **Max** | | **Median** | | | **Mean** | **SD** |
| PhD Advisor relationship (factor) | 1479 | -3.59 | | 0.66 | | 0.44 | | | 0.00 | 0.9600 |
| PhD Belonging, department/social (factor) | 1479 | -3.31 | | 1.16 | | 0.05 | | | 0.00 | 1.0000 |
| PhD Belonging, lab/intellectual (factor) | 1479 | -4.83 | | 0.87 | | 0.52 | | | 0.00 | 1.0000 |
| PhD Faculty support, at institution | 1479 | 1 | | 4 | | 3.00 | | | 3.22 | 0.8100 |
| PhD Faculty support, outside of institution | 1479 | 1 | | 4 | | 3.00 | | | 2.49 | 0.9200 |
| PhD Advisor career advice | 1479 | 1 | | 4 | | 3.00 | | | 3.19 | 0.9600 |
| Years of research prior to PhD program | 1479 | 0 | | 16 | | 2.00 | | | 2.61 | 1.8900 |
| Times supported by NIH (pre-PhD) | 1204 | 0 | | 3 | | 1.00 | | | 0.63 | 0.6400 |
| Top 50 undergraduate institution | 1472 | Yes: 208 | | No: 1264 | | No | | | 0.14 |  |
| **Postdoc and Later Explanatory Variables** |  |  |  | |  | | |  | |  |
| **Independent Variable Predictors of T3 (Current) interest** | **N** | **Min** | **Max** | | **Median** | | | **Mean** | | **SD** |
| Postdoc Advisor relationship (factor) | 1231 | -3.1 | 0.8 | | 0.37 | | | 0.00 | | 0.9600 |
| Postdoc Belonging, department/social (factor) | 1231 | -2.36 | 1.53 | | -0.04 | | | 0.00 | | 1.0000 |
| Postdoc Belonging, lab/intellectual (factor) | 1231 | -3.73 | 0.96 | | 0.41 | | | 0.00 | | 1.0000 |
| Postdoc Faculty support, at institution | 1231 | 1 | 4 | | 3.00 | | | 2.88 | | 1.0000 |
| Postdoc Faculty support, outside of institution | 1231 | 1 | 4 | | 3.00 | | | 2.70 | | 0.9400 |
| Postdoc Advisor career advice | 1182 | 1 | 3 | | 2.00 | | | 2.26 | | 0.7400 |
| Total years of research | 1479 | 4 | 31 | | 12.00 | | | 12.05 | | 3.5100 |
| Times supported by NIH (post-PhD) | 1204 | 0 | 3 | | 1.00 | | | 0.70 | | 0.6800 |
| Years it took to complete PhD | 1479 | 2 | 10 | | 6.00 | | | 5.56 | | 1.0500 |
| Years since completed PhD | 1479 | 0 | 9 | | 5.00 | | | 4.92 | | 2.5700 |
| # of postdoc positions | 1478 | 1 | 5 | | 2.00 | | | 2.03 | | 0.6400 |
| Total time in postdoctoral training | 1241 | 0 | 9 | | 3.00 | | | 3.31 | | 1.8600 |
| First-author publication rate | 1479 | 0 | 7 | | 0.33 | | | 0.41 | | 0.3600 |
| (Career Aspects) Autonomy (factor) | 1479 | -1.47 | 1.84 | | -0.48 | | | 0.00 | | 1.0000 |
| (Career Aspects) Make a difference (factor) | 1479 | -1.55 | 1.39 | | 0.57 | | | 0.00 | | 1.0000 |
| (Career Aspects) Collaboration (factor) | 1479 | -1.16 | 1.81 | | -0.57 | | | 0.00 | | 1.0000 |
| (Career Aspects) Varied work (factor) | 1479 | -1.08 | 1.94 | | -0.53 | | | 0.00 | | 1.0000 |
| (Career Aspects) Ability to do job (factor) | 1479 | -1.23 | 1.71 | | -0.56 | | | 0.00 | | 1.0000 |
| (Career Aspects) Geographic location (factor) | 1479 | -1.23 | 1.68 | | -0.57 | | | 0.00 | | 1.0000 |
| (Career Aspects) Work/Life balance (factor) | 1479 | -1.41 | 1.13 | | 0.78 | | | 0.00 | | 1.0000 |
| (Features of Academia) Funding, Job market, Promotion (factor) | 1479 | -1.86 | 2.92 | | -0.12 | | | 0.00 | | 0.9000 |
| (Features of Academia) Research, Autonomy (factor) | 1479 | -4.16 | 1.36 | | 0.20 | | | 0.00 | | 0.8600 |
| (Features of Academia) Teaching, Mentoring (factor) | 1479 | -3.38 | 1.36 | | 0.13 | | | 0.00 | | 0.8100 |
| (Features of Academia) Work/Life balance (factor) | 1479 | -1.73 | 2.1 | | -0.06 | | | 0.00 | | 0.7200 |
| Confident being independent researcher | 1479 | 1 | 5 | | 4.00 | | | 4.02 | | 1.0800 |
| Top 50 doctoral institution | 1476 | Yes: 770 | No: 706 | | No | | | 0.52 | |  |
