# Supplementary material for: Factors That Influence Career Choice among Different Populations of Neuroscience Trainees
Source: eNeuro. 2021 Jun 18;8(3):ENEURO.0163-21.2021. doi: 10.1523/ENEURO.0163-21.2021 (PMC8223496; doi:10.1523/ENEURO.0163-21.2021)
Supplement: Extended Data Figure 1-1 — Contingency table for gender by current position association. Follow-up analyses performed on significant findings in gender by current position. Effect size: (-) = negligible effect size, (s) = small effect size. * = p < 0.05, ** = p < 0.01. Download Figure 1-1, DOC file. [file enu-eN-SIM-0163-21-s13.doc]

|  | |  |  |  |  |  |
| --- | --- | --- | --- | --- | --- | --- |
| **Current Position** (significance) (effect size) | **Gender** | | | | **Total** | **%** |
|  | **Women** | **%** | **Men** | **%** |  |  |
| Academic Faculty/Research (**) (s) | 179 | 45.2% | 217 | 54.8% | 396 | 100.0% |
| Academic Faculty/Teaching (*) (s) | 65 | 64.4% | 36 | 35.6% | 101 | 100.0% |
| Science/Non-research (*) (s) | 118 | 78.7% | 32 | 21.3% | 150 | 100.0% |
