# Supplementary material for: Factors That Influence Career Choice among Different Populations of Neuroscience Trainees
Source: eNeuro. 2021 Jun 18;8(3):ENEURO.0163-21.2021. doi: 10.1523/ENEURO.0163-21.2021 (PMC8223496; doi:10.1523/ENEURO.0163-21.2021)
Supplement: Extended Data Figure 1-2 — Means for continuous explanatory variables split by gender. Follow-up analyses performed on significant findings in explanatory variables by examining differences in means for subsamples split by gender. N = number in group, M = mean, n = number in subgroup, SD = standard deviation. Effect size: (-) = negligible effect size, (s) = small effect size. * = p < 0.05, ** = p < 0.01, *** = p < 0.001. Download Figure 1-2, DOC file. [file enu-eN-SIM-0163-21-s14.doc]

|  | | | |  |  |  |  |  |  |  |
| --- | --- | --- | --- | --- | --- | --- | --- | --- | --- | --- |
| **Dependent Variable** | **Overall** | | **Gender** | | | | **Mean Diff** | **Pooled SD** | **Cohen's d** |  |
|  |  |  | **Women** | | **Men** | |  |  |  |  |
|  | M | N | M | n | M | n |  |  |  |  |
| PhD Advisor relationship (factor) (***) (s) | 0.00 | 1479 | -0.10 | 793 | 0.12 | 686 | -0.21 | 0.9500 | -0.23 |  |
| PhD Belonging, lab/intellectual (factor) (**) (-) | 0 | 1479 | -0.06 | 793 | 0.07 | 686 | -0.13 | 1 | -0.135 |  |
| PhD Advisor career advice (**) (-) | 3.19 | 1479 | 3.11 | 793 | 3.27 | 686 | -0.16 | 0.9600 | -0.17 |  |
| Postdoc Advisor relationship (factor) (*) (-) | 0.00 | 1231 | -0.07 | 665 | 0.09 | 566 | -0.16 | 0.9600 | -0.17 |  |
| Postdoc Belonging, lab/intellectual (factor) (*) (-) | 0 | 1231 | -0.07 | 665 | 0.08 | 566 | -0.15 | 1 | -0.153 |  |
| Postdoc Advisor career advice (**) (-) | 2.26 | 1182 | 2.20 | 636 | 2.34 | 546 | -0.14 | 0.7400 | -0.19 |  |
| Years since completed PhD (*) (-) | 4.92 | 1479 | 4.74 | 793 | 5.13 | 686 | -0.38 | 2.56 | -0.15 |  |
| First-author publication rate (***) (s) | 0.41 | 1479 | 0.37 | 793 | 0.45 | 686 | -0.09 | 0.3600 | -0.24 |  |
| (Career Aspects) Autonomy (factor) (***) (s) | 0.00 | 1479 | -0.10 | 793 | 0.12 | 686 | -0.22 | 0.9900 | -0.23 |  |
| (Career Aspects) Work/Life balance (factor) (***) (s) | 0.00 | 1479 | 0.13 | 793 | -0.15 | 686 | 0.28 | 0.9900 | 0.28 |  |
| (Features of Academia) Funding, Job market, Promotion (factor) (***) (s) | 0.00 | 1479 | -0.12 | 793 | 0.14 | 686 | -0.26 | 0.8900 | -0.29 |  |
| (Features of Academia) Research, Autonomy (factor) (***) (s) | 0.00 | 1479 | -0.10 | 793 | 0.12 | 686 | -0.23 | 0.8600 | -0.26 |  |
| (Features of Academia) Work/Life balance (factor) (***) (-) | 0.00 | 1479 | -0.06 | 793 | 0.07 | 686 | -0.14 | 0.7100 | -0.20 |  |
| Confident being independent researcher (***) (s) | 4.02 | 1479 | 3.82 | 793 | 4.25 | 686 | -0.42 | 1.0600 | -0.40 |  |
