# Supplementary material for: Factors That Influence Career Choice among Different Populations of Neuroscience Trainees
Source: eNeuro. 2021 Jun 18;8(3):ENEURO.0163-21.2021. doi: 10.1523/ENEURO.0163-21.2021 (PMC8223496; doi:10.1523/ENEURO.0163-21.2021)
Supplement: Extended Data Figure 2-2 — Means for continuous explanatory variables split by UR Status. Follow-up analyses performed on significant findings in explanatory variables by examining differences in means for subsamples split by UR Status. UR = underrepresented, WR = well represented. N = number in group, M = mean, n = number in subgroup, SD = standard deviation. Effect size: (-) = negligible effect size, (s) = small effect size. * = p < 0.05, ** = p < 0.01, *** = p < 0.001. Download Figure 2-2, DOC file. [file enu-eN-SIM-0163-21-s07.doc]

|  | | |  |  |  |  |  | |  | |  | |  | |
| --- | --- | --- | --- | --- | --- | --- | --- | --- | --- | --- | --- | --- | --- | --- |
| **Dependent Variable** | **Overall** | | **UR Status** | | | | | **Mean Diff** | | **Pooled SD** | | **Cohen's d** | |  |
|  |  |  | **WR** | | **UR** | | |  |  |  |  |  |  |  |
|  | M | n | M | n | M | n |  | |  | |  | |  | |
| PhD Belonging, department/social (factor) (*) (-) | 0 | 1479 | 0.03 | 1246 | -0.17 | 233 | 0.2 | | 1 | | 0.205 | |  | |
| PhD Belonging, lab/intellectual (factor) (**) (-) | 0 | 1479 | 0.04 | 1246 | -0.2 | 233 | 0.24 | | 0.99 | | 0.238 | |  | |
| PhD Faculty support, outside of institution (***) (s) | 2.49 | 1479 | 2.45 | 1246 | 2.71 | 233 | -0.26 | | 0.92 | | -0.282 | |  | |
| Postdoc Belonging, lab/intellectual (factor) (***) (s) | 0 | 1231 | 0.05 | 1034 | -0.25 | 197 | 0.3 | | 0.99 | | 0.302 | |  | |
| Postdoc Faculty support, outside of institution (*) (-) | 2.7 | 1231 | 2.67 | 1034 | 2.85 | 197 | -0.18 | | 0.94 | | -0.188 | |  | |
| Years it took to complete PhD (*) (-) | 5.56 | 1479 | 5.53 | 1246 | 5.71 | 233 | -0.18 | | 1.05 | | -0.174 | |  | |
| First-author publication rate (***) (s) | 0.41 | 1479 | 0.42 | 1246 | 0.32 | 233 | 0.1 | | 0.36 | | 0.278 | |  | |
| (Career Aspects) Autonomy (factor) (**) (-) | 0 | 1479 | 0.04 | 1246 | -0.2 | 233 | 0.24 | | 0.99 | | 0.241 | |  | |
| (Features of Academia) Work/Life balance (factor) (**) (-) | 0 | 1479 | -0.03 | 1246 | 0.13 | 233 | -0.16 | | 0.71 | | -0.222 | |  | |
