# Supplementary material for: Factors That Influence Career Choice among Different Populations of Neuroscience Trainees
Source: eNeuro. 2021 Jun 18;8(3):ENEURO.0163-21.2021. doi: 10.1523/ENEURO.0163-21.2021 (PMC8223496; doi:10.1523/ENEURO.0163-21.2021)
Supplement: Extended Data Figure 3-1 — Follow-ups for continuous explanatory variables that had significant interactions of Gender by UR Status. Follow-up analyses performed on significant findings in explanatory variables by examining differences in means for subsamples by interaction between gender and UR Status. UR = underrepresented, WR = well represented. N = number in group, M = mean, n = number in subgroup, SD = standard deviation. Effect size: (-) = negligible effect size, (s) = small effect size. * = p < 0.05, *** = p < 0.001. Download Figure 3-1, DOC file. [file enu-eN-SIM-0163-21-s08.doc]

|  | | | | | | | | |  |
| --- | --- | --- | --- | --- | --- | --- | --- | --- | --- |
| **Dependent Variable / Interaction Context** | **Overall** | | **UR Status** | | | | **Mean Diff** | **Pooled SD** | **Cohen's d** |
|  |  |  | **WR** | | **UR** | |  |  |  |
|  | F | p | M | n | M | n |  |  |  |
| PhD Belonging, lab/intellectual BY UR, within Women (***) (s) | 19.5 | 0 | 0.01 | 660 | -0.43 | 133 | 0.44 | 1.04 | 0.42 |
| PhD Belonging, lab/intellectual BY UR, within Men (-) (-) | 0.05 | 0.8209 | 0.07 | 586 | 0.09 | 100 | -0.02 | 0.92 | -0.025 |
| PhD Belonging, department/social BY UR within Women (***) (s) | 13.74 | 0.0002 | 0.06 | 660 | -0.29 | 133 | 0.35 | 1 | 0.352 |
| PhD Belonging, department/social BY UR within Men (-) (-) | 0.01 | 0.917 | 0 | 586 | -0.01 | 100 | 0.01 | 0.98 | 0.011 |
