# Supplementary material for: Factors That Influence Career Choice among Different Populations of Neuroscience Trainees
Source: eNeuro. 2021 Jun 18;8(3):ENEURO.0163-21.2021. doi: 10.1523/ENEURO.0163-21.2021 (PMC8223496; doi:10.1523/ENEURO.0163-21.2021)
Supplement: Extended Data Figure 4-1 — Omnibus MANOVA means for Change in Career Interest Ratings Over Time. Results from four separate omnibus repeated measures ANOVAs to ascertain whether there were differences in the 4 career interest ratings over time (within-subjects ordinal independent variable). SD = standard deviation. ** = p < 0.01, *** = p < 0.001. Download Figure 4-1, DOC file. [file enu-eN-SIM-0163-21-s09.doc]

|  |  |  |  |  |  |  |  |  |
| --- | --- | --- | --- | --- | --- | --- | --- | --- |
| **Dependent Variables: T2 (End of PhD) Career Interest Ratings** | **Independent Variable: Time** | | | | | | | |
|  | **T1 (Start PhD)** | | **Sig T1 vs T2** | **T2 (End PhD)** | | **Sig T2 vs T3** | **T3 (Current)** | |
|  | Mean | SD |  | Mean | SD |  | Mean | SD |
| Academic Faculty/Research | 3.58 | 0.0345 | *** | 3.23 | 0.0345 | *** | 2.98 | 0.0345 |
| Academic Faculty/Teaching | 2.74 | 0.0354 | *** | 2.58 | 0.0354 | ** | 2.48 | 0.0354 |
| Non-academic Research | 2.50 | 0.0348 | *** | 2.71 | 0.0348 | ** | 2.82 | 0.0348 |
| Science/Non-research | 1.95 | 0.0344 | *** | 2.31 | 0.0344 | *** | 2.48 | 0.0344 |
