# Supplementary material for: Factors That Influence Career Choice among Different Populations of Neuroscience Trainees
Source: eNeuro. 2021 Jun 18;8(3):ENEURO.0163-21.2021. doi: 10.1523/ENEURO.0163-21.2021 (PMC8223496; doi:10.1523/ENEURO.0163-21.2021)
Supplement: Extended Data Figure 5-1 — Follow-ups for significant interactions in Omnibus MANOVA Change in Career Interest Ratings Over Time. Investigating whether Gender or UR Status (between-subjects independent variables) were moderators of differences in the 4 career interest ratings over time. UR = underrepresented, WR = well represented. SD = standard deviation. ** = p < 0.01, *** = p < 0.001. Download Figure 5-1, DOC file. [file enu-eN-SIM-0163-21-s10.doc]

|  | |  |  |  |  |  |  |  |  |  |
| --- | --- | --- | --- | --- | --- | --- | --- | --- | --- | --- |
| **Dependent Variables: T2 (End of PhD) Career Interest Ratings by Interaction Context** | **Levels of Moderator** | **Independent Variable: Time** | | | | | | | | |
|  |  | **T1 (Start PhD)** | | **Sig L1 vs L2** | **T2 (End PhD)** | | **Sig L1 vs L2** | **T3 (Current)** | | **Sig L1 vs L2** |
|  |  | Mean | SD |  | Mean | SD |  | Mean | SD |  |
| Academic Faculty/Research by GENDER by TIME | 1 Women | 3.48 | 0.0455 | *** | 3.08 | 0.0455 | *** | 2.80 | 0.0455 | *** |
|  | 2 Men | 3.69 | 0.0518 |  | 3.38 | 0.0518 |  | 3.15 | 0.0518 |  |
| Academic Faculty/Teaching by UR STATUS by TIME | 1 WR | 2.76 | 0.0279 |  | 2.55 | 0.0279 |  | 2.40 | 0.0279 | * |
|  | 2 UR | 2.72 | 0.0651 |  | 2.61 | 0.0651 |  | 2.55 | 0.0651 |  |
| Science/Non-research by GENDER by TIME | 1 Women | 2.06 | 0.0454 | *** | 2.45 | 0.0454 | *** | 2.70 | 0.0454 | *** |
|  | 2 Men | 1.83 | 0.0517 |  | 2.16 | 0.0517 |  | 2.27 | 0.0517 |  |
