# Supplementary material for: Factors That Influence Career Choice among Different Populations of Neuroscience Trainees
Source: eNeuro. 2021 Jun 18;8(3):ENEURO.0163-21.2021. doi: 10.1523/ENEURO.0163-21.2021 (PMC8223496; doi:10.1523/ENEURO.0163-21.2021)
Supplement: Extended Data Table 2-1 — Correlations for T1->T2 Regressions. Career interest at T2 was regressed on T1 ratings, and correlations with the independent variables were computed with residuals from those procedures (adjusted outcomes). T1 = Time 1 (Start of PhD), T2 = Time 2 (End of PhD). * = p < 0.05, ** = p < 0.01, *** = p < 0.001. Shaded: > 2% variance. Download Table 2-1, DOC file. [file enu-eN-SIM-0163-21-s02.doc]

| **Independent Variable (Graduate School Era Explanatory)** | **Dependent Variable T2/End of Graduate School Career Interest Ratings** (Correlation, Significance) | | | | | | | |
| --- | --- | --- | --- | --- | --- | --- | --- | --- |
|  | **Academic Faculty/Research** | | **Academic Faculty/Teaching** | | **Non-academic Research** | | **Science/Non-research** | |
| PhD Advisor relationship (factor) | 0.2100 | *** | 0.0547 |  | -0.0650 |  | -0.1200 | ** |
| PhD Belonging, department/social (factor) | 0.0800 |  | -0.0055 |  | -0.0220 |  | -0.0170 |  |
| PhD Belonging, lab/intellectual (factor) | 0.2100 | *** | 0.0225 |  | -0.0384 |  | -0.0900 |  |
| PhD Faculty support, at institution | 0.1300 | *** | 0.0198 |  | -0.0423 |  | -0.0160 |  |
| PhD Faculty support, outside of institution | 0.0529 |  | 0.0490 |  | -0.0501 |  | -0.0232 |  |
| PhD Advisor career advice | 0.3100 | *** | 0.1200 | ** | -0.1100 | ** | -0.1500 | *** |
| Years of research prior to PhD program | -0.0109 |  | 0.0047 |  | -0.0309 |  | -0.0716 |  |
| Top 50 undergraduate institution | 0.0133 |  | -0.0297 |  | -0.0082 |  | -0.0277 |  |
| Times supported by NIH (pre-PhD) | 0.0699 |  | 0.0405 |  | -0.0522 |  | 0.0010 |  |
| Have a disability? | -0.0802 |  | -0.0541 |  | 0.0720 |  | 0.0155 |  |
| First person/generation to graduate from 4yr college? | -0.0139 |  | 0.0206 |  | 0.0522 |  | 0.0565 |  |
| Gender | -0.1000 | * | 0.0279 |  | -0.0316 |  | 0.1300 | *** |
| UR Status | -0.0475 |  | 0.0373 |  | 0.0372 |  | 0.1000 | * |
