# Supplementary material for: Factors That Influence Career Choice among Different Populations of Neuroscience Trainees
Source: eNeuro. 2021 Jun 18;8(3):ENEURO.0163-21.2021. doi: 10.1523/ENEURO.0163-21.2021 (PMC8223496; doi:10.1523/ENEURO.0163-21.2021)
Supplement: Extended Data Table 2-2 — Interaction Tests for T1->T2 Regressions. Abbreviated results for investigation of interactions between the graduate school era explanatory variables and gender and UR status. IV = independent variable, T1 = Time 1 (Start of PhD), T2 = Time 2 (End of PhD). * = p < 0.05, ** = p < 0.01, *** = p < 0.001. Download Table 2-2, DOC file. [file enu-eN-SIM-0163-21-s03.doc]

| **Independent Variable  (Graduate School Era Explanatory)** | **Dependent Variable T2/End of PhD Interest Ratings** (Coefficient, Significance) | | | | | | | | | | | | | | | | | | | | | | | | | | |
| --- | --- | --- | --- | --- | --- | --- | --- | --- | --- | --- | --- | --- | --- | --- | --- | --- | --- | --- | --- | --- | --- | --- | --- | --- | --- | --- | --- |
|  | **Academic Faculty/Research** | | | | | | | **Academic Faculty/Teaching** | | | | | | | **Non-academic Research** | | | | | | | **Science/Non-research** | | | | | |
|  | **IV Interaction with …** | | | | | | | **IV Interaction with …** | | | | | | | **IV Interaction with …** | | | | | | | **IV Interaction with …** | | | | | |
|  | **Gender** | | **UR Status** | | | **Gender* UR Status** | | **Gender** | | | **UR Status** | | **Gender* UR Status** | | **Gender** | | **UR Status** | | **Gender* UR Status** | | | **Gender** | | **UR Status** | | **Gender* UR Status** | |
| T1 Interest | -0.0207 |  | | -0.0079 |  | 0.0754 |  | | 0.0414 |  | 0.0214 |  | 0.0776 |  | 0.1280 | ** | -0.0472 |  | | -0.0610 |  | -0.0342 |  | -0.1436 | * | 0.1309 |  |
| PhD Advisor relationship (factor) | -0.0324 |  | | -0.0913 |  | -0.0689 |  | | 0.0172 |  | -0.0518 |  | -0.0830 |  | -0.0442 |  | 0.0331 |  | | 0.0126 |  | 0.0305 |  | 0.0485 |  | 0.1232 |  |
| PhD Belonging, department/social (factor) | -0.0367 |  | | -0.0772 |  | 0.0655 |  | | -0.0020 |  | 0.0234 |  | -0.1257 |  | -0.0117 |  | 0.0277 |  | | -0.1800 |  | -0.0236 |  | 0.0460 |  | -0.2272. |  |
| PhD Belonging, lab/intellectual (factor) | -0.0491 |  | | -0.0370 |  | 0.0483 |  | | -0.0005 |  | 0.0043 |  | -0.0503 |  | 0.0081 |  | 0.0145 |  | | -0.1050 |  | 0.0567 |  | 0.0771 |  | -0.1291 |  |
| Times supported by NIH (pre-PhD) | -0.1061 |  | | -0.1205 |  | 0.0036 |  | | 0.0746 |  | 0.0301 |  | 0.0944 |  | 0.0338 |  | 0.0644 |  | | -0.0388 |  | 0.0704 |  | -0.1829. |  | -0.0547 |  |
| PhD Faculty support, at institution | -0.0632 |  | | -0.0819 |  | -0.0631 |  | | -0.0530 |  | -0.0731 |  | -0.2996 | * | 0.0611 |  | 0.1307 |  | | 0.1200 |  | 0.1059. |  | 0.0155 |  | -0.0932 |  |
| PhD Faculty support, outside of institution | -0.0301 |  | | -0.0072 |  | -0.3206 | * | | 0.0377 |  | -0.0906 |  | -0.0883 |  | 0.0137 |  | 0.0550 |  | | -0.0170 |  | 0.0452 |  | 0.0188 |  | 0.0326 |  |
| PhD Advisor career advice | -0.0131 |  | | -0.0878 |  | -0.1382 |  | | 0.0143 |  | -0.1152 | * | -0.0407 |  | -0.0484 |  | 0.0304 |  | | -0.0021 |  | 0.0287 |  | 0.0530 |  | -0.1867 |  |
| Years of research prior to PhD program | -0.0337 |  | | -0.0200 |  | 0.0213 |  | | -0.0166 |  | -0.0306 |  | 0.0485 |  | -0.0134 |  | -0.0537 |  | | 0.0575 |  | 0.0125 |  | -0.0506 |  | -0.0331 |  |
| Top 50 undergraduate institution | -0.0912 |  | | -0.2088 |  | -0.3470 |  | | 0.1115 |  | 0.0927 |  | -0.4387 |  | -0.1796 |  | 0.1007 |  | | 0.2456 |  | 0.0729 |  | -0.0244 |  | -0.1355 |  |
| Have a disability? | 0.4502 |  | | -0.1048 |  | 0.1651 |  | | 0.2723 |  | 0.3085 |  | 0.1494 |  | 0.2100 |  | -0.1842 |  | | -0.6510 |  | -0.0500 |  | 0.1373 |  | 0.6229 |  |
| First person/ generation to graduate from 4yr college? | -0.0728 |  | | 0.0154 |  | 0.0232 |  | | 0.0985 |  | 0.0644 |  | 0.0034 |  | -0.1562 |  | -0.0896 |  | | -0.1299 |  | 0.0279 |  | 0.0551 |  | 0.1903 |  |
