# Supplementary material for: Factors That Influence Career Choice among Different Populations of Neuroscience Trainees
Source: eNeuro. 2021 Jun 18;8(3):ENEURO.0163-21.2021. doi: 10.1523/ENEURO.0163-21.2021 (PMC8223496; doi:10.1523/ENEURO.0163-21.2021)
Supplement: Extended Data Figure 6-1 — Follow-ups for significant interactions in regressions predicting T2 interest. Follow-up results for significant interactions in the final regressions reported in Table 2. UR = underrepresented, WR = well represented. * = p < 0.05, ** = p < 0.01, *** = p < 0.001. Download Figure 6-1, DOC file. [file enu-eN-SIM-0163-21-s11.doc]

| **Figure 6-1: Follow-ups for significant interactions in regressions predicting T2 interest** | | | | |  |  |
| --- | --- | --- | --- | --- | --- | --- |
| **Dependent Variable: T2 (End of PhD) Career Interest Rating** | **Interaction** | | | | | |
|  | **Gender** | **UR Status** | **Independent Variable** | **Moderator Groups** | **Group Slope** | **Significance of Test of Differences in Slopes** |
| Academic Faculty/Research | Yes | Yes | PhD Faculty support, outside of institution | WR / Women | 0.008 | n.s. |
|  |  |  |  | WR / Men | 0.037 |  |
|  |  |  |  | UR / Women | 0.132 | * |
|  |  |  |  | UR / Men | -0.143 |  |
| Academic Faculty/Teaching | Yes | Yes | PhD Faculty support, at institution | WR / Women | -0.010 | n.s. |
|  |  |  |  | WR / Men | 0.012 |  |
|  |  |  |  | UR / Women | 0.079 | ** |
|  |  |  |  | UR / Men | -0.210 |  |
| Research/Non-academic | Yes | No | T1 interest | Women | 0.536 | ** |
|  |  |  |  | Men | 0.667 |  |
| Science/Non-research | No | Yes | T1 interest | WR | 0.766 | * |
|  |  |  |  | UR | 0.631 |  |
