# Supplementary material for: Factors That Influence Career Choice among Different Populations of Neuroscience Trainees
Source: eNeuro. 2021 Jun 18;8(3):ENEURO.0163-21.2021. doi: 10.1523/ENEURO.0163-21.2021 (PMC8223496; doi:10.1523/ENEURO.0163-21.2021)
Supplement: Extended Data Table 3-1 — Correlations for T3 Regressions. Correlation of all explanatory variables with current interest ratings. * = p < 0.05, ** = p < 0.01, *** = p < 0.001. Shaded: > 2% variance. Download Table 3-1, DOC file. [file enu-eN-SIM-0163-21-s04.doc]

| **Independent Variable (All Explanatory)** | **Dependent Variable**  **T3/Current Career Interest Ratings** (Correlation, Significance) | | | | | | | |
| --- | --- | --- | --- | --- | --- | --- | --- | --- |
|  | **Academic/Research** | | **Academic/Teaching** | | **Non-academic Research** | | **Science/Non-research** | |
| PhD Advisor relationship (factor) | 0.12 | ** | 0.00 |  | 0.01 |  | -0.07 |  |
| PhD Belonging, department/social (factor) | 0.02 |  | 0.00 |  | -0.02 |  | 0.01 |  |
| PhD Belonging, lab/intellectual (factor) | 0.14 | *** | -0.02 |  | 0.00 |  | -0.06 |  |
| PhD Faculty support, at institution | 0.13 | *** | 0.05 |  | 0.02 |  | 0.00 |  |
| PhD Faculty support, outside of institution | 0.11 | * | 0.08 |  | -0.02 |  | -0.02 |  |
| PhD Advisor career advice | 0.30 | *** | 0.10 |  | -0.07 |  | -0.13 | *** |
| Years of research prior to PhD program | -0.01 |  | 0.01 |  | 0.04 |  | -0.02 |  |
| Top 50 undergraduate institution | 0.03 |  | -0.07 |  | 0.01 |  | -0.03 |  |
| Times supported by NIH (pre-PhD) | 0.04 |  | 0.02 |  | -0.04 |  | -0.03 |  |
| Have a disability? | -0.10 | * | -0.06 |  | 0.07 |  | 0.04 |  |
| First person/generation to graduate from 4yr college? | -0.03 |  | 0.03 |  | 0.02 |  | 0.06 |  |
| Gender | -0.15 | *** | 0.02 |  | -0.08 |  | 0.20 | *** |
| UR Status | -0.03 |  | 0.05 |  | 0.03 |  | 0.09 |  |
| Postdoc Advisor relationship (factor) | 0.26 | *** | 0.07 |  | 0.01 |  | -0.14 | *** |
| Postdoc Belonging, department/social (factor) | 0.14 | *** | 0.04 |  | -0.03 |  | -0.11 | * |
| Postdoc Belonging, lab/intellectual (factor) | 0.21 | *** | 0.04 |  | 0.00 |  | -0.15 | *** |
| Postdoc Faculty support, at institution | 0.20 | *** | 0.06 |  | -0.07 |  | -0.11 | * |
| Postdoc Faculty support, outside of institution | 0.16 | *** | 0.04 |  | -0.10 |  | -0.05 |  |
| Postdoc Advisor career advice | 0.35 | *** | 0.11 | * | -0.07 |  | -0.15 | *** |
| Total years of research | 0.14 | *** | -0.01 |  | 0.07 |  | -0.09 |  |
| Top 50 doctoral institution | 0.01 |  | -0.06 |  | 0.00 |  | -0.05 |  |
| Years it took to complete PhD | -0.15 | *** | -0.02 |  | 0.04 |  | 0.12 | ** |
| Years since completed PhD | 0.11 | * | 0.02 |  | -0.09 |  | -0.09 |  |
| # of postdoc positions | 0.13 | *** | 0.05 |  | 0.04 |  | -0.03 |  |
| Total time in postdoctoral training | 0.12 | * | 0.03 |  | -0.06 |  | -0.09 |  |
| First-author publication rate | 0.22 | *** | 0.00 |  | -0.04 |  | -0.14 | *** |
| Times supported by NIH (post-PhD) | 0.03 |  | 0.10 |  | -0.03 |  | 0.04 |  |
| (Career Aspects) Autonomy (factor) | 0.30 | *** | 0.03 |  | -0.12 | ** | -0.15 | *** |
| (Career Aspects) Make a difference (factor) | 0.08 |  | 0.09 |  | -0.03 |  | 0.01 |  |
| (Career Aspects) Collaboration (factor) | 0.17 | *** | -0.01 |  | -0.07 |  | -0.05 |  |
| (Career Aspects) Varied work (factor) | 0.04 |  | 0.01 |  | -0.05 |  | 0.00 |  |
| (Career Aspects) Ability to do job (factor) | 0.14 | *** | 0.10 | * | -0.11 | * | -0.05 |  |
| (Career Aspects) Geographic location (factor) | 0.01 |  | 0.06 |  | 0.04 |  | -0.01 |  |
| (Career Aspects) Work/Life balance (factor) | -0.23 | *** | 0.05 |  | 0.07 |  | 0.15 | *** |
| (Features of Academia) Funding, Job market, Promotion (factor) | 0.53 | *** | 0.19 | *** | -0.18 | *** | -0.27 | *** |
| (Features of Academia) Research, Autonomy (factor) | 0.60 | *** | 0.15 | *** | -0.04 |  | -0.26 | *** |
| (Features of Academia) Teaching, Mentoring (factor) | 0.21 | *** | 0.49 | *** | -0.21 | *** | 0.02 |  |
| (Features of Academia) Work/Life balance (factor) | 0.38 | *** | 0.32 | *** | -0.13 | *** | -0.16 | *** |
| Confident being independent researcher | 0.32 | *** | -0.02 |  | -0.02 |  | -0.22 | *** |
