# Supplementary material for: Factors That Influence Career Choice among Different Populations of Neuroscience Trainees
Source: eNeuro. 2021 Jun 18;8(3):ENEURO.0163-21.2021. doi: 10.1523/ENEURO.0163-21.2021 (PMC8223496; doi:10.1523/ENEURO.0163-21.2021)
Supplement: Extended Data Table 3-2 — Interaction Tests for T3 Regressions. Abbreviated results for investigation of interactions between the explanatory variables and Gender and UR status * = p < 0.05, ** = p < 0.01, *** = p < 0.001. Download Table 3-2, DOC file. [file enu-eN-SIM-0163-21-s05.doc]

| **Independent Variable (Explanatory)** | **Dependent Variable T2/End of Graduate School Career Interest Ratings** (Coefficient, Significance) | | | | | | | | | | | | | | | | | | | | | | | | | | | | | | | | | |
| --- | --- | --- | --- | --- | --- | --- | --- | --- | --- | --- | --- | --- | --- | --- | --- | --- | --- | --- | --- | --- | --- | --- | --- | --- | --- | --- | --- | --- | --- | --- | --- | --- | --- | --- |
|  | **Academic Faculty/Research** | | | | | | | | **Academic Faculty/Teaching** | | | | | | | | | **Non-academic Research** | | | | | | | | | **Science/Non-research** | | | | | | | |
|  | **IV Interaction with …** | | | | | | | | **IV Interaction with …** | | | | | | | | | **IV Interaction with …** | | | | | | | | | **IV Interaction with …** | | | | | | | |
|  | **Gender** | | **UR Status** | | | **Gender*UR Status** | | | **Gender** | | | **UR Status** | | | **Gender*UR Status** | | | **Gender** | | | **UR Status** | | | **Gender*UR Status** | | | **Gender** | | | **UR Status** | | | **Gender*UR Status** | |
| PhD Advisor relationship (factor) | -0.0523 |  | -0.0602 |  | -0.1831 | |  | 0.0167 | |  | -0.0488 | |  | -0.0732 | |  | -0.0391 | |  | 0.0709 | |  | -0.2921 | |  | -0.0279 | |  | 0.0065 | |  | -0.1487 | |  |
| PhD Belonging, department/social (factor) | 0.016 |  | -0.0331 |  | -0.0433 | |  | 0.1005 | |  | 0.0134 | |  | -0.0931 | |  | -0.0064 | |  | 0.0333 | |  | -0.0207 | |  | -0.0107 | |  | 0.0188 | |  | 0.0014 | |  |
| PhD Belonging, lab/intellectual (factor) | -0.0384 |  | -0.0094 |  | 0.0407 | |  | 0.0283 | |  | -0.038 | |  | -0.0078 | |  | 0.0059 | |  | 0.0399 | |  | -0.2256 | |  | 0.0629 | |  | 0.0108 | |  | -0.107 | |  |
| PhD Faculty support, at institution | 0.0526 |  | -0.1066 |  | -0.1465 | |  | 0.0682 | |  | -0.0804 | |  | -0.2887 | |  | 0.0523 | |  | 0.1867 | | . | -0.0392 | |  | 0.1261 | |  | 0.0229 | |  | 0.0215 | |  |
| PhD Faculty support, outside of institution | -0.0563 |  | -0.1238 |  | -0.1751 | |  | 0.0428 | |  | -0.0647 | |  | 0.0343 | |  | -0.0556 | |  | -0.0029 | |  | -0.1402 | |  | 0.0474 | |  | 0.0326 | |  | 0.0937 | |  |
| PhD Advisor career advice | 0.045 |  | -0.1437 |  | -0.1276 | |  | 0.0424 | |  | -0.1849 | | * | 0.0449 | |  | -0.0709 | |  | 0.0189 | |  | -0.0833 | |  | 0.0237 | |  | -0.133 | | . | -0.1773 | |  |
| Years of research prior to PhD program | -0.0545 |  | -0.0478 |  | 0.0273 | |  | 0.0016 | |  | -0.0145 | |  | 0.0256 | |  | -0.0162 | |  | -0.0268 | |  | 0.1248 | |  | 0.0615 | | . | 0.0764 | | . | 0.0964 | |  |
| Undergraduate institution in Top 50 | -0.1235 |  | -0.6032 | . | 0.0113 | |  | 0.3055 | |  | -0.1971 | |  | 0.4236 | |  | -0.1384 | |  | 0.4487 | |  | 0.2229 | |  | 0.0265 | |  | 0.0774 | |  | -1.2763 | | * |
| Times supported by NIH (pre-PhD) | -0.1145 |  | -0.1228 |  | -0.3564 | |  | 0.0089 | |  | 0.0566 | |  | -0.0663 | |  | -0.0158 | |  | 0.1819 | |  | -0.2983 | |  | 0.0056 | |  | -0.1184 | |  | -0.1814 | |  |
| Have a disability? | 0.5554 |  | -0.0553 |  | -0.4893 | |  | 0.0526 | |  | 0.3642 | |  | -0.3194 | |  | 0.1122 | |  | -0.0638 | |  | 0.0138 | |  | -0.4714 | |  | 0.1144 | |  | 0.7524 | |  |
| First person/gen to graduate from 4yr college? | -0.1651 |  | 0.1293 |  | 0.0145 | |  | 0.0056 | |  | 0.2049 | |  | 0.1764 | |  | -0.1567 | |  | -0.1442 | |  | -0.5518 | |  | 0.1516 | |  | 0.1059 | |  | 0.3035 | |  |
| Postdoc Advisor relationship (factor) | -0.041 |  | 0.0443 |  | -0.2107 | |  | -0.0202 | |  | 0.0719 | |  | -0.0913 | |  | -0.0157 | |  | -0.1371 | |  | -0.0281 | |  | 0.0466 | |  | -0.0239 | |  | 0.0706 | |  |
| Postdoc Belonging, department/social (factor) | 0.0697 |  | 0.0414 |  | -0.1254 | |  | 0.0016 | |  | -0.0068 | |  | 0.0569 | |  | 0.0143 | |  | 0.1366 | |  | 0.0613 | |  | -0.0551 | |  | -0.0688 | |  | 0.1809 | |  |
| Postdoc Belonging, lab/intellectual (factor) | -0.0254 |  | 0.0598 |  | -0.0837 | |  | -0.0718 | |  | -0.0059 | |  | 0.0149 | |  | -0.0154 | |  | 0.0421 | |  | -0.2371 | |  | 0.0042 | |  | -0.0327 | |  | 0.0091 | |  |
| Postdoc Faculty support, at institution | -0.1181 | . | -0.0519 |  | -0.1924 | |  | 0.0487 | |  | 0.006 | |  | -0.2375 | |  | 0.0132 | |  | 0.0788 | |  | 0.2314 | |  | 0.0956 | |  | -0.0141 | |  | 0.0928 | |  |
| Postdoc Faculty support, outside of institution | -0.0745 |  | -0.0442 |  | 0.0928 | |  | 0.02 | |  | 0.0317 | |  | 0.0956 | |  | 0.0212 | |  | -0.0917 | |  | 0.053 | |  | 0.0293 | |  | 0.0917 | |  | -0.182 | |  |
| Postdoc Advisor career advice | 0.0144 |  | -0.0897 |  | 0.105 | |  | -0.0765 | |  | -0.0347 | |  | 0.0808 | |  | -0.0744 | |  | 0.0681 | |  | -0.3674 | |  | 0.0684 | |  | 0.0096 | |  | -0.0015 | |  |
| Total years of research | -0.023 |  | -0.0084 |  | 0.0033 | |  | 0.0227 | |  | 0.0314 | |  | 0.0288 | |  | -0.0164 | |  | -0.0195 | |  | 0.043 | |  | 0.0155 | |  | 0.0183 | |  | 0.058 | |  |
| Top 50 doctoral institution | 0.1103 |  | -0.4523 | * | -0.3617 | |  | -0.0535 | |  | -0.2493 | |  | -0.255 | |  | 0.0428 | |  | 0.0198 | |  | 0.3349 | |  | -0.0813 | |  | 0.1385 | |  | -0.3963 | |  |
| Years it took to complete PhD | 0.0611 |  | -0.0671 |  | 0.1005 | |  | 0.0514 | |  | -0.0417 | |  | 0.0379 | |  | 0.0769 | |  | -0.0465 | |  | -0.0701 | |  | -0.1367 | | ** | 0.0458 | |  | 0.0764 | |  |
| Years since completed PhD | 0.0014 |  | -0.0069 |  | 0.0104 | |  | 0.0193 | |  | 0.0383 | |  | 0.0548 | |  | 0.002 | |  | 0.004 | |  | 0.0699 | |  | -0.0212 | |  | 0.009 | |  | 0.0327 | |  |
| # of postdoc positions | -0.0613 |  | 0.1307 |  | 0.2268 | |  | 0.0217 | |  | 0.2015 | |  | 0.0364 | |  | -0.0235 | |  | -0.1305 | |  | 0.0114 | |  | 0.1373 | |  | 0.0079 | |  | 0.1794 | |  |
| Total time in postdoctoral training | -0.0128 |  | 0.0246 |  | -0.0015 | |  | 0.0548 | |  | 0.0808 | |  | 0.0859 | |  | -0.0436 | |  | -0.0068 | |  | -0.0242 | |  | -0.0273 | |  | -0.0247 | |  | 0.0296 | |  |
| First-author publication rate | -0.6141 | *** | 0.6982 | * | 0.3673 | |  | 0.0094 | |  | -0.1624 | |  | 0.2097 | |  | -0.1361 | |  | -0.3559 | |  | -0.2577 | |  | 0.4352 | | * | -0.8002 | | * | 0.0854 | |  |
| Times supported by NIH (post-PhD) | 0.0849 |  | 0.0484 |  | 0.294 | |  | 0.0452 | |  | -0.1083 | |  | -0.1654 | |  | 0.0192 | |  | -0.0127 | |  | -0.1495 | |  | 0.0582 | |  | -0.03 | |  | -0.1218 | |  |

**Table 3-2 (cont.)**

| **Independent Variable (Explanatory)** | **Dependent Variable T2/End of Graduate School Career Interest Ratings** (Coefficient, Significance) | | | | | | | | | | | | | | | | | | | | | | | | |
| --- | --- | --- | --- | --- | --- | --- | --- | --- | --- | --- | --- | --- | --- | --- | --- | --- | --- | --- | --- | --- | --- | --- | --- | --- | --- |
|  | **Academic Faculty/Research** | | | | | | | **Academic Faculty/Teaching** | | | | | | **Non-academic Research** | | | | | | **Science/Non-research** | | | | | |
|  | **IV Interaction with …** | | | | | | | **IV Interaction with …** | | | | | | **IV Interaction with …** | | | | | | **IV Interaction with …** | | | | | |
|  | **Gender** | | **UR Status** | | | **Gender*UR Status** | | **Gender** | | **UR Status** | | **Gender*UR Status** | | **Gender** | | **UR Status** | | **Gender*UR Status** | | **Gender** | | **UR Status** | | **Gender*UR Status** | |
| (Career Aspects) Autonomy (factor) | 0.0169 |  | | -0.0032 |  | -0.2021 |  | 0.0126 |  | 0.0394 |  | -0.0752 |  | 0.0001 |  | 0.0052 |  | 0.0004 |  | 0.0902 |  | 0.0674 |  | 0.0169 |  |
| (Career Aspects) Make a difference (factor) | 0.028 |  | | 0.0619 |  | -0.0602 |  | 0.0177 |  | -0.0359 |  | -0.3067 |  | 0.0881 |  | -0.0111 |  | 0.105 |  | 0.023 |  | 0.1119 |  | -0.0364 |  |
| (Career Aspects) Collaboration (factor) | 0.0221 |  | | -0.0614 |  | 0.0315 |  | 0.0842 |  | 0.0649 |  | -0.2927 |  | -0.0018 |  | -0.0685 |  | -0.1411 |  | 0.0587 |  | 0.1379 | . | -0.0297 |  |
| (Career Aspects) Varied work (factor) | -0.0215 |  | | -0.0805 |  | -0.1779 |  | 0.0532 |  | -0.0109 |  | -0.1835 |  | 0.0227 |  | 0.0246 |  | -0.055 |  | 0.0479 |  | 0.0391 |  | -0.1955 |  |
| (Career Aspects) Ability to do job (factor) | 0.0308 |  | | -0.059 |  | 0.1067 |  | 0.0816 |  | 0.0396 |  | -0.2017 |  | -0.0377 |  | 0.0072 |  | -0.2737 |  | 0.0391 |  | 0.1139 |  | -0.1189 |  |
| (Career Aspects) Geographic location (factor) | -0.0555 |  | | 0.1692 | . | -0.0539 |  | -0.0018 |  | -0.0075 |  | -0.164 |  | 0.0123 |  | -0.0632 |  | -0.2379 |  | -0.0465 |  | -0.1243 |  | -0.1661 |  |
| (Career Aspects) Work/Life balance (factor) | 0.0822 |  | | 0.0887 |  | -0.022 |  | -0.01 |  | 0.0165 |  | 0.0834 |  | 0.0974 |  | 0.0192 |  | -0.0304 |  | -0.0922 |  | -0.0847 |  | -0.0731 |  |
| (Features of Academia) Funding, Job market, Promotion (factor) | -0.1044 | . | | -0.0163 |  | 0.0113 |  | -0.0003 |  | 0.0746 |  | 0.0965 |  | -0.1029 |  | 0.0757 |  | -0.01 |  | 0.1668 | ** | 0.0225 |  | 0.0344 |  |
| (Features of Academia) Research, Autonomy (factor) | -0.0602 |  | | -0.0232 |  | 0.2296 |  | 0.0327 |  | 0.0453 |  | -0.0425 |  | -0.1427 | * | -0.0762 |  | -0.1497 |  | 0.1768 | ** | -0.0361 |  | -0.1202 |  |
| (Features of Academia) Teaching, Mentoring (factor) | -0.0276 |  | | 0.1047 |  | 0.1799 |  | -0.0037 |  | -0.0921 |  | 0.0529 |  | 0.0341 |  | -0.0475 |  | -0.3779 | . | 0.1044 |  | 0.0446 |  | -0.1403 |  |
| (Features of Academia) Work/Life balance (factor) | -0.1534 | . | | 0.0306 |  | -0.0135 |  | -0.0257 |  | 0.1091 |  | 0.2224 |  | -0.0744 |  | 0.0274 |  | -0.1657 |  | 0.2195 | ** | 0.0486 |  | -0.112 |  |
| Confident being independent researcher | -0.0296 |  | | -0.0289 |  | -0.2088 |  | -0.0447 |  | 0.0828 |  | -0.0768 |  | -0.0036 |  | -0.0193 |  | 0.1696 |  | 0.0562 |  | -0.1217 | . | 0.3541 | * |
