# Supplementary material for: Factors That Influence Career Choice among Different Populations of Neuroscience Trainees
Source: eNeuro. 2021 Jun 18;8(3):ENEURO.0163-21.2021. doi: 10.1523/ENEURO.0163-21.2021 (PMC8223496; doi:10.1523/ENEURO.0163-21.2021)
Supplement: Extended Data Figure 11-1 — Follow-ups for significant interactions in regressions predicting T3 interest. Follow-up results for significant interactions in the final regressions reported in Table 3. UR = underrepresented, WR = well represented. * = p < 0.05, ** = p < 0.01, *** = p < 0.001. Download Figure 11-1, DOC file. [file enu-eN-SIM-0163-21-s12.doc]

|  | | | | |  |  |
| --- | --- | --- | --- | --- | --- | --- |
| **Dependent Variable: T3 (Current) Career Interest Rating** | **Interaction** | | | | | |
|  | **Gender** | **UR Status** | **Independent Variable** | **Moderator Groups** | **Group Slope** | **Significance of Test of Differences in Slopes** |
| Academic Faculty/Teaching | No | Yes | PhD advisor career advice | WR | 0.034 | * |
|  |  |  |  | UR | -0.107 |  |
| Science/Non-research | Yes | Yes | Top 50 undergraduate institution | Not / WR / Women | 2.49 | *** |
|  |  |  |  | Not / WR / Men | 2.23 |  |
|  |  |  |  | Not / UR / Women | 2.53 | n.s. |
|  |  |  |  | Not / UR / Men | 2.49 |  |
|  |  |  |  | Top 50 / WR / Women | 2.38 | n.s. |
|  |  |  |  | Top 50 / WR / Men | 2.24 |  |
|  |  |  |  | Top 50 / UR / Women | 2.68 | ** |
|  |  |  |  | Top 50 / UR / Men | 1.36 |  |
|  | Yes | No | Years it took to complete PhD | Women | 0.120 | ** |
|  |  |  |  | Men | -0.043 |  |
|  | Yes | Yes | Confident being independent researcher | WR / Women | -0.049 | n.s. |
|  |  |  |  | WR / Men | -0.132 |  |
|  |  |  |  | UR / Women | -0.258 | * |
|  |  |  |  | UR / Men | 0.070 |  |
|  | No | Yes | First-author publication rate | WR | -0.029 | * |
|  |  |  |  | UR | -0.744 |  |
|  | Yes | No | Research, Autonomy (factor) | Women | -0.127 | * |
|  |  |  |  | Men | 0.051 |  |
